# Poly(2-oxazoline)-Based Thermoresponsive Stomatocytes

**DOI:** 10.1021/acs.biomac.4c00726

**Published:** 2024-08-15

**Authors:** Roberto Terracciano, Yuechi Liu, Zivani Varanaraja, Magdalena Godzina, Gokhan Yilmaz, Jan C. M. van Hest, C. Remzi Becer

**Affiliations:** † Department of Chemistry, University of Warwick, Coventry CV4 7AL, U.K.; ‡ Eindhoven University of Technology, P.O. Box 513, Eindhoven 5600MB, The Netherlands

## Abstract

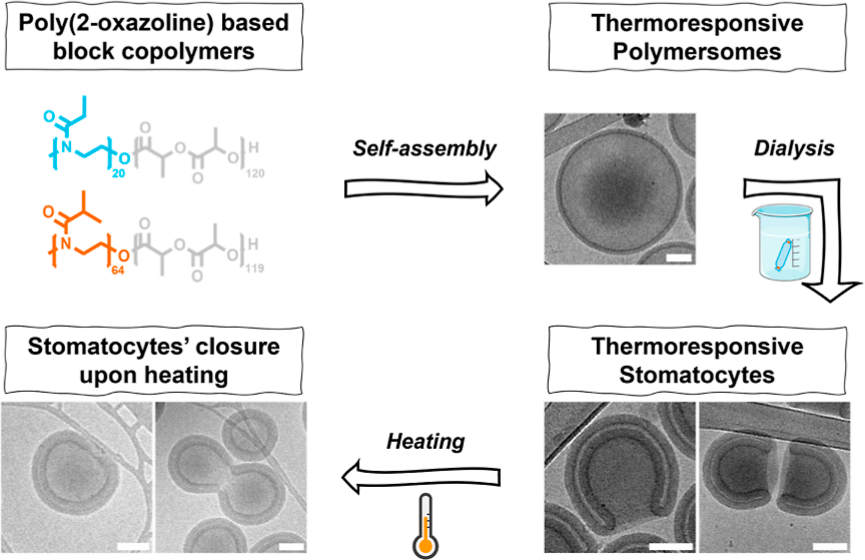

The design of biocompatible and biodegradable nanostructures with controlled morphological features remains a predominant challenge in medical research. Stimuli-responsive vesicles offer significant advantages in drug delivery, biomedical applications, and diagnostic techniques. The combination of poly(2-oxazoline)s with biodegradable polymers could provide exceptional biocompatibility properties and be proposed as a versatile platform for the development of new medicines. Therefore, poly(2-ethyl-2-oxazoline) (PEtOx) and poly(2-isopropyl-2-oxazoline) (P*i*PrOx) possessing a hydroxy terminal group that acts as an initiator for the ring-opening polymerization of d,l-lactide (DLLA) have been utilized in this study. The resulting amphiphilic block polymers were used to create polymersomes, which undergo solvent-dependent reorganization into bowl-shaped vesicles or stomatocytes. By blending PEtOx-*b*-PDLLA and P*i*PrOx-*b*-PDLLA copolymers, a thermoresponsive stomatocyte was generated, where the opening narrowed and irreversibly closed with a slight increase in the temperature. Detailed transmission electron microscopy analysis reveals the formation of both closed and fused stomatocytes upon heating the sample above the critical solution temperature of P*i*PrOx.

## Introduction

Considering the excellent capability of amphiphilic block copolymers to be used as building blocks for nanoparticles in biomedical applications, it is crucial to investigate novel strategies for synthesizing amphiphilic macromolecules that demonstrate self-assembly behavior.^1,2^ In particular, self-assembled polymeric vesicles, or polymersomes, are a versatile class of nanovehicles, because of their loading capacity with both hydrophilic and hydrophobic cargo and their ability to change shape, which enables tunability of particle topology for the interaction with living cells. Among these topologies, bowl-shaped polymersomes or stomatocytes have been studied extensively, with particular focus on those based on poly(ethylene glycol)-*b*-poly(d,l-lactide) due to their biocompatibility.^3^ Poly(lactides) have emerged as valuable materials in biomedical engineering owing to their degradation properties.^4−7^ Poly(ethylene glycol) is the hydrophilic domain of choice because of its stealth properties. However, considering the potential future placement on the market as a medicinal product, it is important to note that a growing segment of the population exhibits an immunogenic response to PEG, sparking interest in finding a viable substitute.^8^

Poly(2-oxazoline)s (POxs) constitute a class of polymeric materials that has undergone extensive research for a diverse array of potential applications, with a specific focus on their importance in the biomedical field.^9−11^ In fact, POxs are largely employed for medical and biological applications (such as drug delivery systems or bioinspired sensors) as they are biocompatible and demonstrate predominantly nontoxic attributes.^12,13^ In particular, POxs have garnered interest as potential substitutes for PEG since their discovery that coating liposomes with poly(2-methyl-2-oxazoline) (PMeOx) and poly(2-ethyl-2-oxazoline) (PEtOx) showed comparable efficacy to PEG in prolonging circulation times.^14,15^ This makes them viable candidates for supplantation in therapeutic contexts.^16^ Although their use in vitro and in vivo remains relatively limited in the literature, the combination of polylactides and POx as block copolymers, bearing or encapsulating targeting ligands, expands the repertoire of active drug delivery strategies.^17^ Examples in the literature demonstrate that PEtOx-*b*-PLA micelles encapsulating temoporfin significantly reduced skin phototoxicity in vivo within a photodynamic therapy (PDT)-mediated cancer therapy context.^18^ Additionally, other studies showcased their potential in combating multidrug resistance (MDR) as well as acting as antimicrobial materials.^19,20^

Thermoresponsive nanoparticles change their properties in response to a change in the temperature. They hold promise in various areas such as nanomotion,^21^ modulation of membrane permeability^22^ to enhance drug release for cancer treatment^23,24^ or combating infections,^25^ and driving self-assembly.^26−28^ Belonging to the class of POxs is poly(2-isopropyl-2-oxazoline) (P*i*PrOx), endowed with a lower critical solution temperature (LCST) behavior in water. As the LCST of P*i*PrOx is close to body temperature, this polymer has developed into the most abundantly studied and popular thermoresponsive POx derivative, making it an optimal candidate for developing smart materials whose solubility is influenced by the temperature. Nevertheless, the thermal responsiveness of linear P*i*PrOx may vary when this polymer is organized into alternative molecular architectures, polymer composition, or nanoparticle structures.^29−32^ Nanoparticles triggered at around 40–42 °C can be engineered to release drugs specifically in areas with slightly elevated temperatures, such as inflamed tissues or tumors.^33^ This targeted release can be finely adjusted based on various temperature ranges and formulations.^34^

In this study, we present the transformation of spherical polymersomes, consisting of PEtOx-PDLLA block copolymers, into precisely defined stomatocytes through exposure to osmotic pressure at low temperatures. In a biological context, offering new alternative structures is crucial not only for vectors encapsulating drugs but also for morphologies that significantly influence cellular uptake.^35^ For instance, red blood cell (RBC)-derived vesicles have been investigated as potential biocompatible gene delivery vectors. These vesicles aim to enhance cargo delivery by increasing their circulation time through evasion of macrophage uptake.^36^ Utilizing a similar “artificial cell” principle, which mimics the shape of natural cells, offers valuable insights. These principles contribute to minimizing immune recognition, enhancing circulation duration, and ultimately improving therapeutic efficacy in nanomedicine advancements.

**Figure 1:**
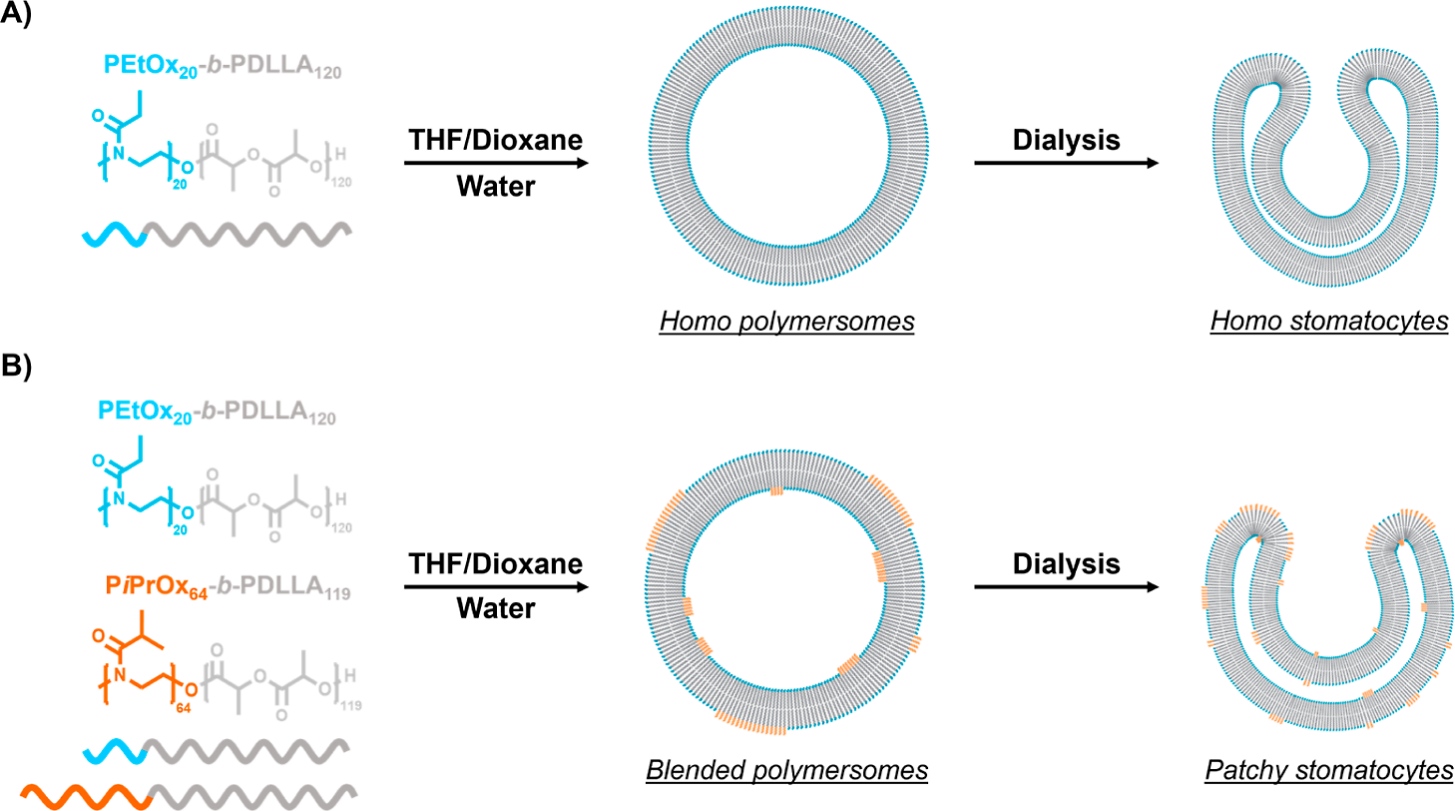
(A) Illustration depicting the formation of polymersomes and their osmotic-induced shape transformation into stomatocytes from poly(2-ethyl-2-oxazoline)-*b*-poly(d,l-lactide). (B) Schematic of stomatocyte formation by coblending poly(2-ethyl-2-oxazoline)-*b*-poly(d,l-lactide) with poly(2-isopropyl-2-oxazoline)-*b*-poly(d,l-lactide) copolymers. Based on our hypothesis, the P*i*PrOx-PDLLA polymers primarily cluster within specific regions, corresponding to the edges of the stomatocytes.

Additionally, by utilizing P*i*PrOx-PDLLA diblock copolymers, we tailored the nanostructures to exhibit an LCST near physiological conditions. Subsequently, we explored the impact of temperature increase on the morphology of this system. Enhancing the potential of nanoparticles in therapeutic applications may involve optimizing their formulation and creating more intricate self-assembled structures. This approach could complement existing treatments while reducing off-target toxicities. This study represents the first example of creating POx-based stomatocytes whose morphology can be effectively controlled in a temperature-tunable manner (Figure 1).

## Experimental Section

### Materials

All chemicals were used as received unless otherwise stated. 2-Ethyl-2-oxazoline 99+% (Acros Organics, EtOx) was dried over calcium hydride and distilled under reduced pressure prior to use. 2-Isopropyl-2-oxazoline was synthesized as previously reported.^37^ Methyl *p*-toluenesulfonate 98% (Aldrich, PropTos) was distilled under reduced pressure and stored under nitrogen. Extra-dry acetonitrile 99+% was purchased from Acros Organics and stored over molecular sieves under an inert atmosphere. d,l-Lactide was purchased from Sigma-Aldrich and used as supplied. All other chemicals were supplied by Sigma-Aldrich and were used without any purification. Ultra-pure Milli-Q water was obtained from a Merck Millipore Q-Pod system (18.2 MΩ) and a 0.22 μm Millipore Express 40 filter was used for the self-assembly of polymersomes and their dialysis. Spectra/Por MWCO 12–14,000 g mol^–1^ dialysis membranes were used for dialysis during stomatocyte formation. Sodium chloride was purchased from Merck.

### Methods

#### ^1^H Nuclear Magnetic Resonance

All spectra were recorded on a Bruker ADVANCE III HD 400 MHz. CDCl_3_ was used as the solvent. Data analysis was performed by using MestReNova software.

#### Gel Permeation Chromatography

The measurements were performed by using THF (2% TEA and 0.01% BHT) as the eluent. The Agilent Technologies 1260 Infinity instrument was equipped with refractive index (RI) and 308 nm UV detectors, a PLgel 5 μm guard column, and a PLgel 5 μm mixed D column (300 × 7.5 mm). Samples were run at 1 mL min^–1^ at 40 °C. Poly(methyl methacrylate) standards (Agilent PMMA calibration kits, M-M-10 and M-L-10 MW range 500–120,000) were used for the calibration. Before injection (100 μL), the samples were filtered through a PTFE membrane with a 0.2 μm pore size. The data was determined by conventional calibration using Agilent gel permeation chromatography/size exclusion chromatography (GPC/SEC) software and plotted in OriginPro 2022b.

#### Matrix-Assisted Laser Desorption Ionization Time-of-Flight Mass Spectrometry

All matrix-assisted laser desorption ionization time-of-flight mass spectrometry (MALDI-ToF MS) analyses were performed on a Bruker Autoflex Speed mass spectrometer by using a nitrogen laser at 337 nm with positive ion detection. Polymer samples were prepared as follows: solutions of *trans*-2-[3-(4-tertbutylphenyl)-2-methyl-2-propylidene]malonitrile (DCTB, ≥98%) as the matrix (30 mg/mL in THF), sodium trifluoroacetate (NaTFA) as the cationization agent (1 mg/mL in ethanol), and sample (5 mg/mL in THF) were mixed in a volume ratio of 5:2:5 and then spotted onto the target (0.5 μL). Spectra were recorded in reflective mode, and the mass spectrometer was calibrated with poly(methyl methacrylate) standards.

#### Differential Scanning Calorimetry

Thermal transitions were determined on a Mettler Toledo DSC1 equipped with an autosampler under a nitrogen atmosphere with a flow of 50 mL min^–1^. 40 μL aluminum pans with 5–15 mg of the sample were prepared. Initial ramp was from 25 to 200 °C at 50 °C min^–1^, followed by quenching to −100 °C at −150 °C min^–1^. Then, two heating and cooling cycles from −50 to 200 °C at a rate of 5 °C min^–1^ were performed. The *T*_g_ value was calculated using Mettler Toledo thermal analysis software, identified as a second-order exothermic transition in the second heating curve.

#### UV–Vis Spectroscopy

An Agilent Technologies Cary 100 UV–Vis spectrophotometer equipped with an Agilent Technologies Cary temperature controller and an Agilent Technologies 6 × 6 multicell block Peltier was used for the turbidity analyses for determining the cloud point temperatures (*T*_cp_) of each sample. The measurements were performed using Suprasil quartz cuvettes (Hellman, 100-QS, light path of 10.00 mm) filled with 5 mg mL^–1^ of each polymer in 150 mM NaCl aqueous solution. Two heating and cooling cycles between 25 and 60 °C were performed with a temperature gradient of 1 °C min^–1^ at λ = 520 nm for each sample. All of the data were obtained using Cary WinUV software and plotted using OriginPro 2021b. Cloud point temperatures (*T*_cp_) were determined at 50% transmittance.

#### Dynamic Light Scattering

Measurements were carried out on an Anton Paar Litesizer. Samples were measured at 25 °C at a backscattering measuring angle of 175°. Each sample was measured in triplicate with 30 runs per measurement and 5 min equilibration time between each measurement. For temperature-dependent dynamic light scattering (DLS) data, the samples were analyzed three times at every temperature. The heating rate was 1 °C, with 5 min equilibration time.

#### Cryogenic Transmission Electron Microscopy

A 5 μL aliquot of the formulation was pipetted onto a carbon-coated copper grid (HC300Cu, Holey Carbon film on Copper 300 mesh, EM Resolutions, UK). Excess solution was blotted away with filter paper, and the grid was plunge-frozen in liquid ethane and cooled by liquid nitrogen. The sample was then maintained at liquid nitrogen temperatures throughout the analysis with a Gatan 914 cryo-holder. Transmission electron microscopy (TEM) images were taken on a JEOL 2100Plus TEM instrument at a voltage of 200 kV using a Gatan OneView IS digital camera.

### Synthetic Procedures

#### Synthesis of 2-Isopropyl-2-oxazoline

2-Isopropyl-2-oxazoline (*i*PrOx) was synthesized by following a previously reported procedure.^37^ Briefly, isobutyronitrile (1 equiv) and zinc acetate dihydrate (catalyst, 0.02 equiv) were heated to 130 °C under reflux, and 2-aminoethanol (1.1 equiv) was added dropwise. After the overnight reaction, the mixture was cooled to room temperature; dichloromethane was added, and the organic phase was washed three times with water and once with brine. Dichloromethane was then removed under reduced pressure, and the monomer was purified by fractional distillation over ninhydrin, followed by distillation from barium oxide.

#### Synthesis of POx Homopolymers

All homopolymers were synthesized by cationic ring-opening polymerization (CROP) under similar conditions, varying the reaction time depending on the monomer and the degree of polymerization (DP) being used. The polymerizations were performed with an initial monomer concentration of 4 M in MeCN using methyl tosylate as the initiator. As an example, the CROP of PEtOx_20_-OH was synthesized as follows. EtOx (2.00 g, 20.18 mmol, 20 equiv), methyl tosylate (0.19 g, 1.009 mmol), and MeCN (5.04 mL) were transferred into a sealed microwave vial equipped with a magnetic stirrer bar. The microwave vial was preheated and sealed before transfer to remove any contamination and water. After the reaction mixture was transferred, it was degassed with nitrogen for 30 min. A small aliquot was taken to calculate [M]/[I] via ^1^H nuclear magnetic resonance (NMR). The vial was then placed in an oil bath and the reaction proceeded for 60 min at 100 °C. After 60 min, the vial was lifted from the oil bath, and a relief needle was inserted into the seal to release pressure from the vial. Potassium hydroxide (5.04 mmol, 0.28 g, 5 equiv) in methanol was immediately added to terminate the reaction. After being stirred for 2 h at 25 °C, the reaction mixture was diluted using DCM and washed twice with brine. The organic phase was then dried over MgSO_4_. After filtration, the solvent was removed under reduced pressure, and the polymer precipitated into cold ether to yield the product PEtOx_20_-OH (1.60 g, white powder).

#### Synthesis of POx_n_-PDLLA_m_ Diblock Copolymers

All diblock copolymers were synthesized by ring-opening polymerization (ROP) according to a modified literature procedure,^3,4^ varying the macroinitiators and the DP being used. As an example, the ROP of PEtOx_20_-PDLLA_120_ was synthesized as follows. PEtOx_20_-OH macroinitiator (0.20 g, 0.1 mmol) was weighed into a round-bottom flask along with d,l-lactide (1.73 g, 12 mmol, 120 equiv) in order to achieve a PDLLA chain with 120 repeat units. Dry toluene (ca. 3× 50 mL) was then added to the flask and the solvent was evaporated to dry the contents before polymerization. The dried reagents were then redissolved in dry DCM (24 mL, [monomer] = 0.5 M) and DBU was added (3 equiv with respect to the macroinitiator; 0.3 mmol = 44.8 μL) under nitrogen. The reaction proceeded at rt for around 2 h, until there was no evidence of the monomer from the ^1^H NMR spectrum. After completion was confirmed by ^1^H NMR, the reaction mixture was diluted using DCM and washed twice with 1 M KHSO_4_ and once with brine before drying with MgSO_4_, filtering, and evaporating most of the solvent. The concentrated copolymer solution (in DCM) was then precipitated into ice-cold diethyl ether (100 mL) and the product was dried under vacuum to yield a white powder (1.45 g). Copolymer composition was calculated by comparing the protons of POxs (1.04–1.18 ppm) to PDLLA CH (multiplet at 5.09–5.26 ppm). The average polymer compositions and values for molecular weights, PDI and *T*_g_, are given in Table 1.

**Table 1:** Characterization of POx Homopolymers and Poly(2-oxazoline)-*b*-poly(d,l-lactide) Diblock Copolymers

polymer	*M*_n,theo_ (g/mol)	*M*_n,GPC_ (g/mol)	D̵	*T*_g_ (°C)
PEtOx_20_	2000	2000	1.10	43
PEtOx_30_	3000	2900	1.14	51
P*i*PrOx_64_	7300	7300	1.08	79
PEtOx_20_-*b*-PDLLA_120_	17,900	17,700	1.15	53
PEtOx_20_-*b*-PDLLA_83_	15,700	11,800	1.11	53
PEtOx_30_-*b*-PDLLA_130_	21,700	16,100	1.24	55
P*i*PrOx_64_-*b*-PDLLA_119_	23,300	18,300	1.26	54

#### Preparation of Polymersomes

In a 15 mL vial, PEtOx_*n*_-PDLLA_*m*_ (10 mg) alone or a mixture of PEtOx_20_-PDLLA_120_ and P*i*PrOx_64_-PDLLA_119_ block copolymers (4:1 w/w, 10 mg) were dissolved in 1 mL of THF and dioxane (1:4 v/v), and the vial was sealed with a rubber septum. After complete dissolution of the polymers, the solution was stirred at 750 rpm prior to the addition of Milli-Q (1 mL, 1 mL h^–1^) via a syringe pump. A needle was inserted into the septum to release pressure.

#### General Method for Stomatocytes Preparation

First, a batch of polymersomes was prepared following the previously mentioned procedure. The resulting cloudy suspension was transferred into a prehydrated dialysis bag (SpectraPor, MWCO: 12–14 kDa, 2 mL/cm). Polymersomes were dialyzed against precooled salt solution (75 mM NaCl, unless stated otherwise) at 5 °C for a maximum of 24 h with a water change after 1 h.

## Results and Discussion

### Synthesis and Characterization of Hydroxyl-Terminated POx Macroinitiators and Subsequent Poly(2-oxazoline)-*b*-poly(d,l-lactide) Block Copolymers

**Figure 2:**
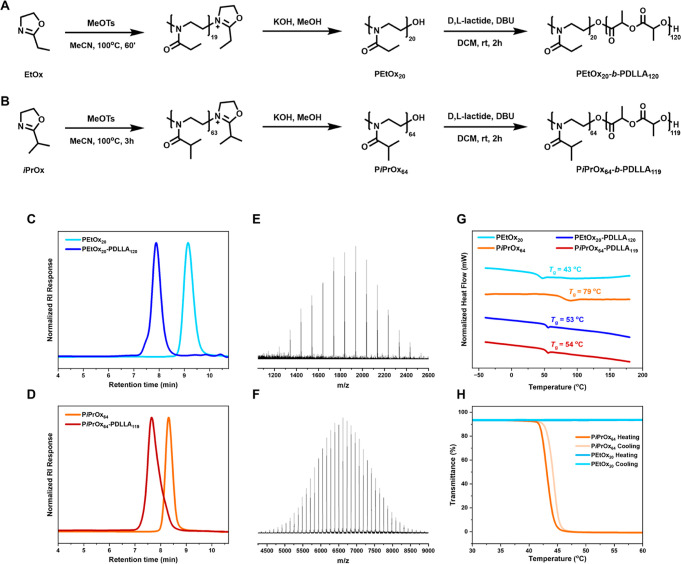
(A) Synthesis of PEtOx and (B) P*i*PrOx through CROP. Both polymers were terminated by direct end capping with potassium hydroxide post-CROP. Following purification, chain extension was achieved by ROP catalyzed by DBU using d,l-lactide as the monomer, resulting in the formation of PEtOx_20_-PDLLA_120_ and P*i*PrOx_64_-PDLLA_119_. (C) GPC traces of PEtOx_20_ and (D) P*i*PrOx_64_, along with their respective PDLLA diblock copolymers. (E) MALDI-ToF spectra of PEtOx_20_ and (F) P*i*PrOx_64_. The distributions correspond to the sodium adduct of the hydroxy-terminated polymers. (G) DSC thermograms of the second heating cycle of PEtOx_20_, P*i*PrOx_64_, PEtOx_20_-PDLLA_120_, and P*i*PrOx_64_-PDLLA_119_. Glass transition temperatures were calculated from the inflection point of the curves. (H) Turbidity measurements of the second heating and cooling cycles of PEtOx_20_ and P*i*PrOx_64_ (5 mg/mL) in Milli-Q water (150 mM NaCl) conducted at λ = 520 nm.

The synthesis of PEtOx involved employing monomer-to-initiator ratios [M]/[I] = 20 or 30 at 100 °C to ensure a better control over the polymerization and chain-end fidelity.^38^ For the synthesis of P*i*PrOx, a monomer-to-initiator ratio [M]/[I] = 70 was utilized at the same polymerization temperature. The relatively lower polymerization temperature enabled the immediate end-capping reaction with KOH in methanol. Methyl tosylate (MeTos) acted as the initiator for all of the polymerizations. The synthetic procedures for all homopolymers are outlined in Figure 2A,B. Each polymerization reaction achieved complete conversion within 60 min for PEtOx_20_, 90 min for PEtOx_30_, and 92% conversion in 3 h for P*i*PrOx_64_. To ensure that the polymers possessed a hydroxyl group at the ω-end to serve as macroinitiators, all polymerization reactions were terminated with an excess of potassium hydroxide and then purified (see the Experimental Section). End-group analysis via MALDI-ToF MS of each homopolymer displayed a single distribution corresponding to the sodium adduct of DP_20_ and DP_30_ PEtOx and DP_64_ P*i*PrOx, indicating complete end-group functionalization (Figures 2E,F and S11). Although DP_30_ PEtOx and DP_64_ P*i*PrOx show a minor secondary distribution, these can be attributed to the hydrogen-initiated chains.^39,40^ A difference of 14 Da, corresponding to the mass of methylene (−CH_2_−), separates the major distribution and minor distribution (Na adduct) of both PEtOx_30_ and P*i*PrOx_64_. Within each spectrum of PEtOx, the distribution peaks were separated by 99 Da, corresponding to the mass of the EtOx repeat unit (Figure S11). For P*i*PrOx, the peaks were separated by 113 Da, corresponding to the mass of the *i*PrOx repeat unit. Taking PEtOx_20_ as an example, the theoretical mass of the sodium adduct of the functionalized polymer with KOH is 2036.68 Da, while the observed mass is 2036.81 Da, with a difference of 0.13 Da. End-group verification for all macroinitiators is available in the Supporting Information. GPC traces of DP_20–30_ PEtOx and DP_64_ P*i*PrOx indicated monomodal molecular weight distributions with narrow dispersity values ranging from 1.08 to 1.14 (Figures 2C,D and S10 and Table 1). The equilibrium nature of the ROP of the lactide, combined with the effectiveness of POx as the macroinitiator, enabled the maintenance of relatively low polydispersity indices, ranging from 1.11 to 1.26, upon extension of the polylactide segment. A clear shift to higher molecular weights observed by GPC and ^1^H NMR analysis of all copolymers validated the successful chain extension of PEtOx and P*i*PrOx with d,l-lactide. This was evidenced by the appearance of peaks at 1.6–1.4 and 5.2–5.0 ppm corresponding to −CH_3_ and −CH of the lactide block, respectively. Nonetheless, a slight overlap between P*i*PrOx_64_ and its corresponding diblock was observed within the 8–8.5 min range in GPC. This observation, coupled with the escalating polydispersity values as both molecular weights of the macroinitiators and the length of the lactide segment increase, indicates that smaller molecular weight macroinitiators exhibit greater efficiency due to their higher reactivity and reduced chain entanglement. Furthermore, it is noteworthy to highlight that the elongation of d,l-lactide chains with PEtOx and P*i*PrOx as macroinitiators, along with DBU as the organocatalyst at room temperature, presents a metal-free method for generating poly(2-oxazoline)-poly(d,l-lactide) diblock copolymers without dependence on coupling reactions^41−43^ or elevated temperatures and reaction times.^44−46^

The thermal properties of the PEtOx-PDLLA and P*i*PrOx-PDLLA copolymers, alongside their respective POx homopolymers, were assessed via differential scanning calorimetry (DSC) (Figure 2G). PEtOx_20/30_ displayed glass transition temperatures (*T*_g_) of 43 and 51 °C, respectively, notably lower than the reported *T*_g_ value for PEtOx with higher degrees of polymerization (*T*_g_ = 60–62 °C).^47,48^ This discrepancy may stem from variations in chain length, as proposed by the Flory–Fox model, which posits that *T*_g_ increases with molecular weight.^49^ Conversely, P*i*PrOx_64_ exhibited a *T*_g_ around 79 °C, in good agreement with the literature (66–70 °C).^45,50−52^ Additionally, DSC analysis was conducted on POx-PDLLA diblock copolymers, all of which displayed similar *T*_g_ values of 53–55 °C (Table 1). The temperature closer to the *T*_g_ of PDLLA homopolymers (45–50 °C)^45,53,54^ can be attributed to the longer poly(d,l-lactide) chains disrupting oxazoline backbones packing and preventing strong interaction with amide dipoles. Moreover, the reason PDLLA affects the *T*_g_ of PEtOx and P*i*PrOx in opposite directions may lie in the chain conformation and packing and how the PDLLA influences the crystallinity of the copolymers. When PDLLA induces a more ordered structure, the *T*_g_ can increase, as seen with the PEtOx-PDLLA copolymer where the ratio of PDLLA to PEtOx is higher than that to P*i*PrOx. Conversely, if the poly(d,l-lactide) disrupts the crystallinity of P*i*PrOx, the *T*_g_ might decrease, as observed. We hence conclude that the impact on crystallinity can differ between PEtOx and P*i*PrOx due to their unique side chain structures and packing behavior in the presence of PDLLA in their respective diblock copolymers.

Next, the behavior of PEtOx and P*i*PrOx in aqueous solution was evaluated through turbidity measurements. Solutions of 5 mg mL^–1^ concentration were prepared and subjected to two heating/cooling cycles from 25 to 60 °C, monitored at a wavelength of 520 nm. Figure 2H shows the second heating and cooling cycles of both polymers in an aqueous solution of 150 mM of NaCl. At room temperature, both polymers are fully soluble in water; with increasing temperature, no change in turbidity was detected for PEtOx. This observation aligns with literature findings indicating that PEtOx homopolymers with a DP less than 100 do not exhibit a cloud point between 0 and 100 °C.^55^ Conversely, there was a noticeable change in turbidity with an increasing temperature for the P*i*PrOx solution. The transmittance decreases, indicating a cloud point temperature (*T*_cp_) of ∼43 °C, which was determined at 50% transmittance and is not significantly different from previously reported values.^56^

### Stomatocytes Shape Transformation from Poly(2-ethyl-2-oxazoline)-*b*-poly(d,l-lactide) Copolymers

The block copolymers PEtOx_20_PDLLA_83/120_ and PEtOx_30_PDLLA_130_ were further utilized for self-assembly. Following the solvent switch methodology outlined previously,^3^ copolymer solutions of 10 mg/mL in THF/dioxane (1:4, v/v) were diluted with water up to 50% v/v at a rate of 1 mL/h with stirring. Subsequently, the resultant nanoparticles were immediately transferred to a dialysis bag and dialyzed against a saline solution (75 mM NaCl) at 5 °C.

Previous studies have elucidated the critical role of organic solvent within the inner compartment of the nanoparticle in plasticizing the membrane, thereby facilitating shape transformation induced by disparity in osmotic pressure across the membrane.^4^ This solvent expulsion persists until the membrane loses its flexibility, returning to a glassy state and thereby stabilizing the vesicle structure.

**Figure 3:**
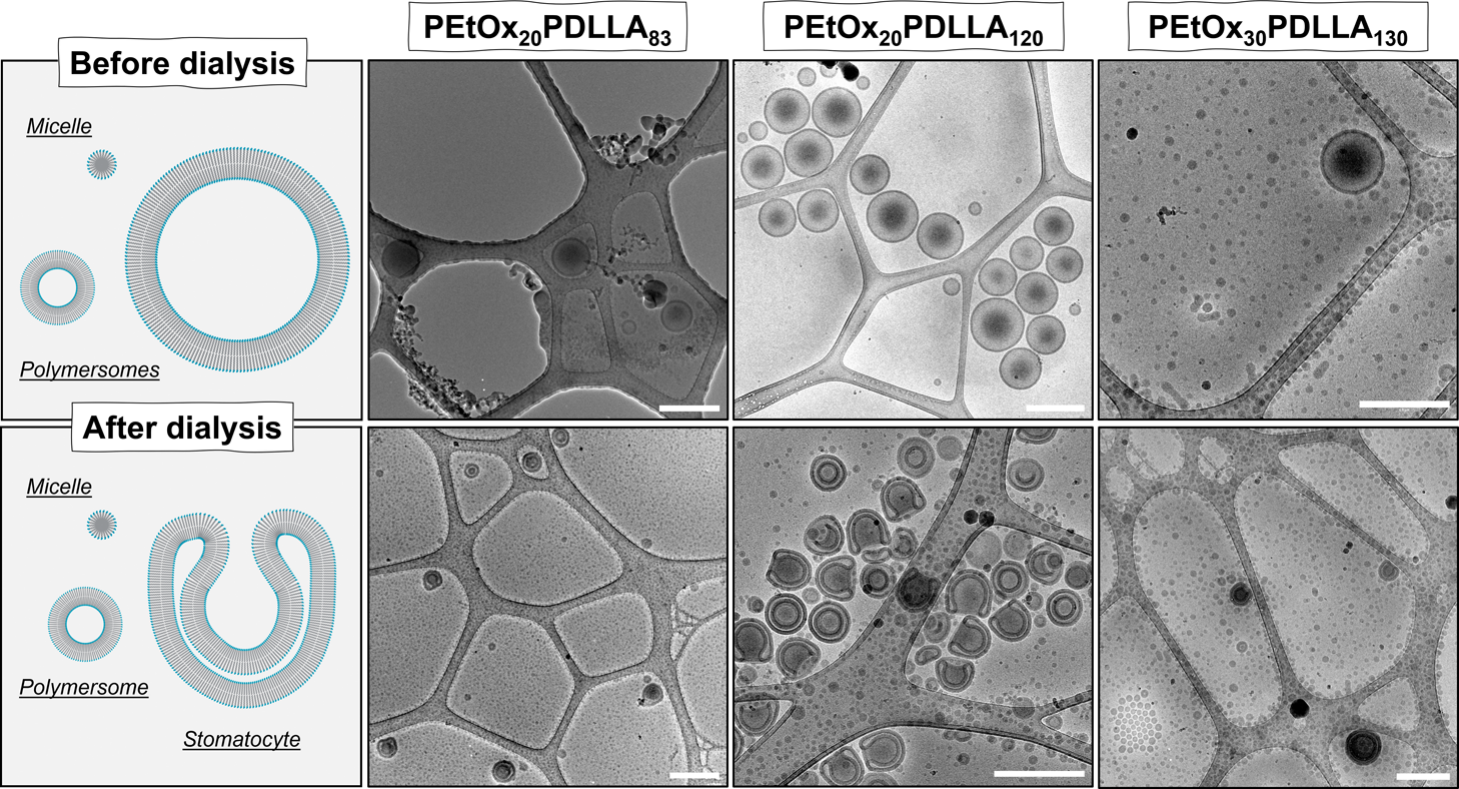
Cryo-TEM images of PEtOx_20_-*b*-PDLLA_83/120_ and PEtOx_30_-*b*-PDLLA_130_ vesicles before and after dialysis in 75 mM NaCl aqueous solution (scale bars = 500 nm).

The copolymers PEtOx_20_-PDLLA_83_ and PEtOx_30_-PDLLA_130_ predominantly yielded micelles of 15–40 nm and relatively small polymersomes of approximately 300–450 nm in diameter, which underwent shape transformation to stomatocytes upon dialysis, reaching around 140 nm in size (Figure 3). The nearly identical hydrophilic mass fractions of *f*_PEtOx_ in these two copolymers (13 and 14%, respectively) are consistent. However, maintaining the DP of PEtOx at 20 and increasing the hydrophobic to hydrophilic ratio by elongating the PDLLA block to a DP of 120, resulting in a mass fraction *f*_PEtOx_ = 10%, led to an increased yield of polymersomes in both quantity and size. Following self-assembly, the nanostructures exhibited an average diameter of 600 nm with a moderate size distribution (PDI of 0.09), as determined by DLS measurements (Figure S15). Cryogenic TEM (cryo-TEM) analysis further confirmed the presence of spherical polymer vesicles (Figures 3 and S19). Upon dialysis against a 75 mM saline aqueous solution, a reduction of approximately 66% in the average diameter of the polymersomes to 200 nm was observed, with no alterations in the size distribution (PDI of 0.09) (Figure S15). Cryo-TEM images provided additional evidence for the formation of stomatocytes, the yield of which was higher than those obtained from PEtOx_20_-PDLLA_83_ and PEtOx_30_-PDLLA_130_. Particles formed from these two polymers are typically organized into smaller polymersomes or micelles. These structures are not large enough to accommodate organic solvents in their core, and their membrane surface area is insufficient to induce bending into transformed nanoparticles. Consequently, as observed for both PEtOx_20_-PDLLA_83_ and PEtOx_30_-PDLLA_130_, these structures do not undergo any bending deformations and remain unchanged upon solvent switch by dialysis.

To facilitate shape transformation, understanding the energetic state of a polymersome membrane, characterized by its bending energy (*E*_b_),^57^ is crucial. Influenced by factors such as temperature, solvent composition, and membrane asymmetry, *E*_b_ can be minimized through two energetic pathways under osmotic pressure: deflation via prolates (toward tubes) or oblates (resulting in discs and stomatocytes). The negative contribution of spontaneous curvature (*C*_0_) favors oblates, pivotal in determining shape transformations’ direction, possibly due to a mismatch between the external and internal membrane surfaces. While outside this study’s scope, further investigation into PEtOx-PDLLA properties is warranted to understand the contribution of *C*_0_ in shape transformations, as well as the effect of the hydrophilic/hydrophobic ratio on self-assembly and subsequent membrane curvature.

### Thermally Triggered Morphological Evolution of Stomatocytes

P*i*PrOx, a thermoresponsive pseudopeptide, shows LCST-type phase behavior near human body temperature, making it highly attractive for numerous bioapplications.^58−61^ Regulating the dynamic flexibility of membrane curvature and the directional transformation of vesicle morphology is essential for the development of a self-assembled system with lifelike properties. Various customized transformation behaviors have been documented in response to distinct external triggers.^62−65^

In fabricating nanoarchitectures, copolymer blending has proven to be an effective design strategy.^3^ Here, we opted to maintain the hydrophobic component constant (DP 120) while increasing the length of the P*i*PrOx. The decision was guided by the need to strike a balance, aiming for a polymer with an appropriate length capable of initiating the ring opening of d,l-lactide, thus enabling its incorporation into a stomatocyte formulation. Additionally, the chosen length needed to sufficiently lower the LCST of the system to a value near body temperature while remaining smaller than the glass transition (*T*_g_) of two diblocks (∼54 °C).

As a starting point for formulation, we blended PEtOx-PDLLA: P*i*PrOx-PDLLA with identical PDLLA block lengths at a w/w ratio of 4:1, while also exploring formulations with lower concentrations of P*i*PrOx-PDLLA. However, we proceeded with the aforementioned ratio, driven by the need to adjust factors, including concentration, to potentially further lower the LCST of the nanostructure. As described previously, we followed the solvent switch methodology applying the same conditions. The blended polymers were dissolved in THF/dioxane (1:4, v/v) and then diluted with water. Subsequently, the resulting nanoparticles were dialyzed against a saline solution (75 mM NaCl) at 5 °C. Our observations revealed the formation of stomatosomal structures measuring approximately 200 nm (Figure S20). In contrast, P*i*PrOx_64_-PDLLA_119_, when formulated alone, resulted in micelles and aggregates (Figure S17). The membrane thickness of the formed structures was measured to be 23 nm, not significantly different from the 21 nm of the stomatocyte membranes formulated solely with PEtOx_20_-PDLLA_120_ (Figure S18).

**Figure 4:**
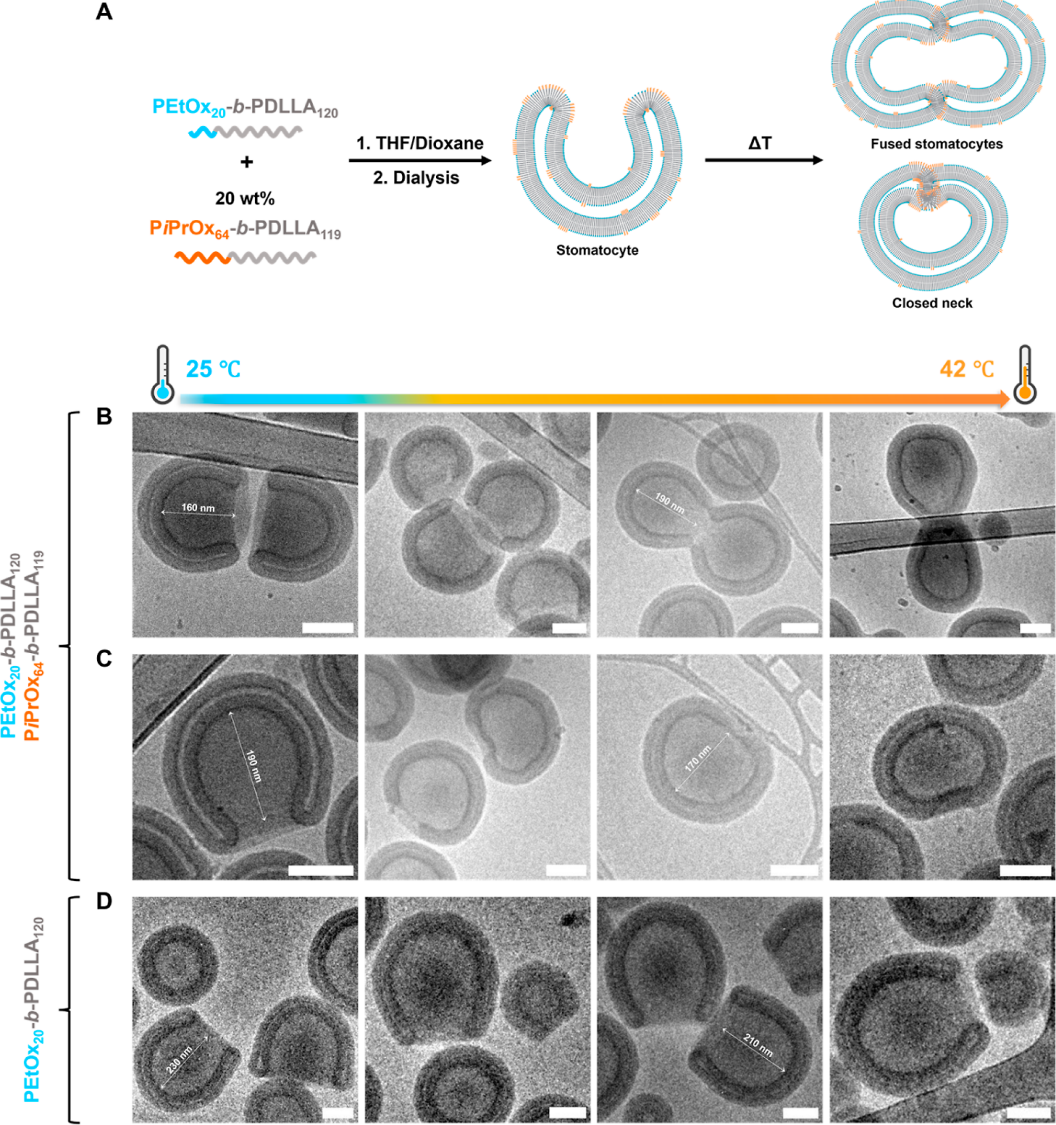
(A) Schematic temperature-dependent evolution (40–42 °C) of stomatocytes’ fusion and neck closing. (B) Temperature-dependent morphological change visualized by cryogenic scanning electron microscopy of stomatocytes facing each other and (C) single stomatocyte (10 mg/mL in 150 mM NaCl aqueous solution). The first two stacked images on the left depict the stomatocytes in their postformation state, before exposure to temperature. (D) PEtOx_20_-*b*-PDLLA_120_-based stomatocytes do not show any fluctuations in size or morphology upon heating. All scale bars correspond to 100 nm.

Next, the effect of temperature on the size and morphology of stomatocytes containing P*i*PrOx_64_-PDLLA_119_ was investigated by DLS and cryo-TEM. At ambient temperature (25 °C) and below the LCST, the stomatocytes exhibited their typical bowl shape of 200 nm in size. Upon heating to 42 °C, the size increased to approximately 340 nm, as investigated by temperature-dependent DLS (Figure S24). Further examination by cryo-TEM allowed us to gain more insights into the shapes of the particles and elucidate the evolution of their morphology (Figure 4). For this purpose, the sample was heated at temperatures between 40 and 42 °C for 5 min and immediately frozen. It was observed that individual stomatocytes gradually underwent structural alteration, resulting in the irreversible closure of the stoma. This convergence of the edges upon heating resulted in the fusion of two stomatocytes when they were oriented with the apertures facing each other. The majority of stomatocytes appeared to be closed in the area of the neck, with almost no instances of ubiquitous aggregation observed (Figure S22). Additionally, the gradual development of a distinct fluctuating area on the stomatocytes edges is observable (Figure S23). This phenomenon may indicate the presence of a phase-separated region, likely caused by the abundance of isopropyl chains becoming insoluble in water. The driving force for the phase separation and the consequent closing of the neck is attributed tentatively to the release of water molecules bound to the P*i*PrOx chains and the improved polymer–polymer interaction of *i*PrOx above LCST. We propose the existence of a “sweet spot” where, if promptly cooled down, the nanoparticles’ shape would revert to their original form. However, the P*i*PrOx blocks undergo thermo-induced aggregation and crystallization, even when the solution is not exposed to heat for prolonged periods or temperatures far above the LCST.^66,67^ The morphology observed in this temperature-responsive system is similar to what was previously described with structures known as “nested vesicles”,^68^ although these particles are generally obtained through changes in osmotic stresses. Furthermore, stomatocytes containing only PEtOx_20_-PDLLA_120_ were used as a negative control and did not exhibit any changes in size or morphology upon heating. This confirms that the incorporation of P*i*PrOx_64_-PDLLA_119_ is responsible for creating temperature-tunable stomatocytes (Figure S21).

Our hypotheses attempt to address this phenomenon. The first hypothesis posits that, to minimize bending energy, the polymers distribute themselves symmetrically within oblate vesicles. Near the poles, where the local mean curvature is small, components with a smaller spontaneous curvature tend to reside. Conversely, at the equator, where the local mean curvature is larger, components with larger spontaneous curvature predominate.^69^ This distribution results in an asymmetrical arrangement, with one component’s domains situated at the poles, separated by the other component’s domain along the equator. The former is represented by P*i*PrOx_64_-PDLLA_119_, whereas the latter is represented by the PEtOx_20_-PDLLA_120_. However, it is worth noting that specific values for the intrinsic curvature are not known. In contrast to the first hypothesis, one can speculate that, if the spontaneous curvatures of the membrane components differ substantially, the components tend to mix to minimize the bending energy. In this conjecture, it is the change in temperature that leads to the demixing and separation of the P*i*PrOx_64_-PDLLA_119_ polymers, causing them to diffuse to the stomatocytes’ edges and form domains.

The thermal switch at ∼40 to 42 °C is particularly relevant for drug delivery systems used in gene therapy and cancer treatment.^33,34^ This temperature range is critical for hyperthermia-triggered drug release treatments in vivo, typically occurring between 39 and 45 °C.^34,70,71^ One prominent example is ThermoDox, a heat-activated liposomal formulation currently in Phase III clinical trials for cancer treatment, which operates effectively at 39.5–42 °C. Further research is needed to optimize hyperthermia-based treatments for patients. Combining hyperthermia with targeted therapy agents, immunotherapy, nanomedicine, or particle therapy shows great promise for future clinical applications. Therefore, developing novel multimembrane nanoparticle platforms is warranted, as they may offer significant advantages in these contexts. In summary, thermoresponsive nanoparticles, used in conjunction with local hyperthermia, hold great potential to enhance therapeutic outcomes, improving efficacy and reducing side effects.

## Conclusions

The first examples of POx-based stomatocytes with tunable morphology and size by temperature have been demonstrated. By blending PEtOx_20_-*b*-PDLLA_120_ with P*i*PrOx_64_-*b*-PDLLA_119_, stomatocytes gradually underwent morphological changes, resulting in either the irreversible closure of the neck or the fusion of two stomatocytes when oriented with the apertures facing each other as the temperature increased. Using DLS, cryo-TEM, and theoretical considerations, we propose and discuss a model that accounts for the observed phenomena. In biomedical applications and clinical settings, advanced stimuli-responsive nanomaterials that integrate diverse functionalities with biocompatibility are paramount. Thermoresponsive nanoparticles show significant potential in temperature-triggered drug release, local hyperthermia treatment, and gene therapy, highlighting their versatility and promise in enhancing drug delivery and therapeutic strategies through localized temperature-sensitive responses. The ability of stomatocytes to reorganize into more complex self-assembled and fused structures upon heating can significantly impact their biological relevance. This morphological transformation can alter biodistribution, cellular uptake profiles, and drug release characteristics, allowing for on-demand tuning of these features where needed. Exploring various combinations of these materials can potentially lead to the development of new multimembrane nanoparticle platforms that outperform their single-membrane counterparts, leveraging these characteristics to fully realize their potential in medical practice.

## Supporting Information Available

Detailed experimental data, synthesis procedures, NMR spectra, MALDI-ToF data, GPC traces, DSC data, DLS data, and cryo-TEM images (PDF)

## Notes

Published as part of Biomacromolecules special issue “Stimuli-Responsive Polymers at the Interface with Biology”.

## References

[ref1] Cui W.; Li J.; Decher G. Self-Assembled Smart Nanocarriers for Targeted Drug Delivery. Adv. Mater. 2016, 28 (6), 1302–1311. 10.1002/adma.201502479. 26436442

[ref2] Ge W.; Wang L.; Zhang J.; Ou C.; Si W.; Wang W.; Zhang Q.; Dong X. Self-Assembled Nanoparticles as Cancer Therapeutic Agents. Adv. Mater. Interfaces 2021, 8 (1), 2001602 10.1002/admi.202001602.

[ref3] Pijpers I. A. B.; Abdelmohsen L. K. E. A.; Williams D. S.; van Hest J. C. M. Morphology Under Control: Engineering Biodegradable Stomatocytes. ACS Macro Lett. 2017, 6 (11), 1217–1222. 10.1021/acsmacrolett.7b00723. 29214115 PMC5708263

[ref4] Abdelmohsen L. K. E. A.; Williams D. S.; Pille J.; Ozel S. G.; Rikken R. S. M.; Wilson D. A.; van Hest J. C. M. Formation of Well-Defined, Functional Nanotubes via Osmotically Induced Shape Transformation of Biodegradable Polymersomes. J. Am. Chem. Soc. 2016, 138 (30), 9353–9356. 10.1021/jacs.6b03984. 27374777 PMC4974604

[ref5] Wauters A. C.; Scheerstra J. F.; Vermeijlen I. G.; Hammink R.; Schluck M.; Woythe L.; Wu H.; Albertazzi L.; Figdor C. G.; Tel J.; et al. Artificial Antigen-Presenting Cell Topology Dictates T Cell Activation. ACS Nano 2022, 16 (9), 15072–15085. 10.1021/acsnano.2c06211. 35969506 PMC9527792

[ref6] Pijpers I. A. B.; Cao S.; Llopis-Lorente A.; Zhu J.; Song S.; Joosten R. R. M.; Meng F.; Friedrich H.; Williams D. S.; Sánchez S.; et al. Hybrid Biodegradable Nanomotors through Compartmentalized Synthesis. Nano Lett. 2020, 20 (6), 4472–4480. 10.1021/acs.nanolett.0c01268. 32427492 PMC7291354

[ref7] Shao J.; Cao S.; Williams D. S.; Abdelmohsen L. K. E. A.; van Hest J. C. M. Photoactivated Polymersome Nanomotors: Traversing Biological Barriers. Angew. Chem., Int. Ed. 2020, 59 (39), 16918–16925. 10.1002/anie.202003748. PMC7540338 32533754

[ref8] Chen B.-M.; Cheng T.-L.; Roffler S. R. Polyethylene Glycol Immunogenicity: Theoretical, Clinical, and Practical Aspects of Anti-Polyethylene Glycol Antibodies. ACS Nano 2021, 15 (9), 14022–14048. 10.1021/acsnano.1c05922. 34469112

[ref9] Beyer V. P.; Monaco A.; Napier R.; Yilmaz G.; Becer C. R. Bottlebrush Glycopolymers from 2-Oxazolines and Acrylamides for Targeting Dendritic Cell-Specific Intercellular Adhesion Molecule-3-Grabbing Nonintegrin and Mannose-Binding Lectin. Biomacromolecules 2020, 21 (6), 2298–2308. 10.1021/acs.biomac.0c00246. 32320219

[ref10] Blakney A. K.; Abdouni Y.; Yilmaz G.; Liu R.; McKay P. F.; Bouton C. R.; Shattock R. J.; Becer C. R. Mannosylated Poly(ethylene imine) Copolymers Enhance saRNA Uptake and Expression in Human Skin Explants. Biomacromolecules 2020, 21 (6), 2482–2492. 10.1021/acs.biomac.0c00445. 32250603

[ref11] Zahoranová A.; Luxenhofer R. Poly(2-oxazoline)- and Poly(2-oxazine)-Based Self-Assemblies, Polyplexes, and Drug Nanoformulations—An Update. Adv. Healthcare Mater. 2021, 10 (6), 2001382 10.1002/adhm.202001382. PMC11468752 33448122

[ref12] Luxenhofer R.; Sahay G.; Schulz A.; Alakhova D.; Bronich T. K.; Jordan R.; Kabanov A. V. Structure-property relationship in cytotoxicity and cell uptake of poly(2-oxazoline) amphiphiles. J. Controlled Release 2011, 153 (1), 73–82. 10.1016/j.jconrel.2011.04.010. PMC3134160 21513750

[ref13] Luxenhofer R.; Han Y.; Schulz A.; Tong J.; He Z.; Kabanov A. V.; Jordan R. Poly(2-oxazoline)s as Polymer Therapeutics. Macromol. Rapid Commun. 2012, 33 (19), 1613–1631. 10.1002/marc.201200354. 22865555 PMC3608391

[ref14] Woodle M. C.; Engbers C. M.; Zalipsky S. New Amphipatic Polymer-Lipid Conjugates Forming Long-Circulating Reticuloendothelial System-Evading Liposomes. Bioconjugate Chem. 1994, 5 (6), 493–496. 10.1021/bc00030a001. 7873652

[ref15] Zalipsky S.; Hansen C. B.; Oaks J. M.; Allen T. M. Evaluation of Blood Clearance Rates and Biodistribution of Poly(2-oxazoline)-Grafted Liposomes§. J. Pharm. Sci. 1996, 85 (2), 133–137. 10.1021/js9504043. 8683436

[ref16] Bauer M.; Lautenschlaeger C.; Kempe K.; Tauhardt L.; Schubert U. S.; Fischer D. Poly(2-ethyl-2-oxazoline) as Alternative for the Stealth Polymer Poly(ethylene glycol): Comparison of in vitro Cytotoxicity and Hemocompatibility. Macromol. Biosci. 2012, 12 (7), 986–998. 10.1002/mabi.201200017. 22648985

[ref17] Stafast L. M.; Engel N.; Görls H.; Weber C.; Schubert U. S. End-functionalized diblock copolymers by mix and match of poly(2-oxazoline) and polyester building blocks. Eur. Polym. J. 2023, 184, 111779 10.1016/j.eurpolymj.2022.111779.

[ref18] Shieh M.-J.; Peng C.-L.; Chiang W.-L.; Wang C.-H.; Hsu C.-Y.; Wang S.-J. J.; Lai P.-S. Reduced Skin Photosensitivity with meta-Tetra(hydroxyphenyl)chlorin-Loaded Micelles Based on a Poly(2-ethyl-2-oxazoline)-b-poly(d,l-lactide) Diblock Copolymer in Vivo. Mol. Pharm. 2010, 7 (4), 1244–1253. 10.1021/mp100060v. 20469890

[ref19] Zhao Y.; Zhou Y.; Wang D.; Gao Y.; Li J.; Ma S.; Zhao L.; Zhang C.; Liu Y.; Li X. pH-responsive polymeric micelles based on poly(2-ethyl-2-oxazoline)-poly(d,l-lactide) for tumor-targeting and controlled delivery of doxorubicin and P-glycoprotein inhibitor. Acta Biomater. 2015, 17, 182–192. 10.1016/j.actbio.2015.01.010. 25612838

[ref20] Concilio M.; Garcia Maset R.; Lemonche L. P.; Kontrimas V.; Song J.-I.; Rajendrakumar S. K.; Harrison F.; Becer C. R.; Perrier S. Mechanism of Action of Oxazoline-Based Antimicrobial Polymers Against Staphylococcus aureus: In Vivo Antimicrobial Activity Evaluation. Adv. Healthcare Mater. 2023, 12 (29), 2301961 10.1002/adhm.202301961. PMC11468764 37522292

[ref21] Tu Y.; Peng F.; Sui X.; Men Y.; White P. B.; van Hest J. C. M.; Wilson D. A. Self-propelled supramolecular nanomotors with temperature-responsive speed regulation. Nat. Chem. 2017, 9 (5), 480–486. 10.1038/nchem.2674. 28430193

[ref22] Kim C.-J.; Ercole F.; Chen J.; Pan S.; Ju Y.; Quinn J. F.; Caruso F. Macromolecular Engineering of Thermoresponsive Metal-Phenolic Networks. J. Am. Chem. Soc. 2022, 144 (1), 503–514. 10.1021/jacs.1c10979. 34958559

[ref23] Agarwal A.; Mackey M. A.; El-Sayed M. A.; Bellamkonda R. V. Remote Triggered Release of Doxorubicin in Tumors by Synergistic Application of Thermosensitive Liposomes and Gold Nanorods. ACS Nano 2011, 5 (6), 4919–4926. 10.1021/nn201010q. 21591812

[ref24] Kashyap S.; Singh N.; Surnar B.; Jayakannan M. Enzyme and Thermal Dual Responsive Amphiphilic Polymer Core-Shell Nanoparticle for Doxorubicin Delivery to Cancer Cells. Biomacromolecules 2016, 17 (1), 384–398. 10.1021/acs.biomac.5b01545. 26652038

[ref25] Qing G.; Zhao X.; Gong N.; Chen J.; Li X.; Gan Y.; Wang Y.; Zhang Z.; Zhang Y.; Guo W.; et al. Thermo-responsive triple-function nanotransporter for efficient chemo-photothermal therapy of multidrug-resistant bacterial infection. Nat. Commun. 2019, 10 (1), 4336 10.1038/s41467-019-12313-3. 31551496 PMC6760232

[ref26] Nishimura T.; Shishi S.; Sasaki Y.; Akiyoshi K. Thermoresponsive Polysaccharide Graft Polymer Vesicles with Tunable Size and Structural Memory. J. Am. Chem. Soc. 2020, 142 (27), 11784–11790. 10.1021/jacs.0c02290. 32506909

[ref27] Nishimura T.; Hatatani Y.; Ando M.; Sasaki Y.; Akiyoshi K. Single-component nanodiscs via the thermal folding of amphiphilic graft copolymers with the adjusted flexibility of the main chain. Chem. Sci. 2022, 13 (18), 5243–5251. 10.1039/D2SC01674E. 35655565 PMC9093194

[ref28] Hirayama S.; Oohora K.; Uchihashi T.; Hayashi T. Thermoresponsive Micellar Assembly Constructed from a Hexameric Hemoprotein Modified with Poly(N-isopropylacrylamide) toward an Artificial Light-Harvesting System. J. Am. Chem. Soc. 2020, 142 (4), 1822–1831. 10.1021/jacs.9b10080. 31904965

[ref29] Amirova A. I.; Dudkina M. M.; Tenkovtsev A. V.; Filippov A. P. Self-assembly of star-shaped poly(2-isopropyl-2-oxazoline) in aqueous solutions. Colloid Polym. Sci. 2015, 293 (1), 239–248. 10.1007/s00396-014-3402-x.

[ref30] Zhang N.; Luxenhofer R.; Jordan R. Thermoresponsive Poly(2-oxazoline) Molecular Brushes by Living Ionic Polymerization: Kinetic Investigations of Pendant Chain Grafting and Cloud Point Modulation by Backbone and Side Chain Length Variation. Macromol. Chem. Phys. 2012, 213 (9), 973–981. 10.1002/macp.201200015.

[ref31] Huber S.; Hutter N.; Jordan R. Effect of end group polarity upon the lower critical solution temperature of poly(2-isopropyl-2-oxazoline). Colloid Polym. Sci. 2008, 286 (14–15), 1653–1661. 10.1007/s00396-008-1942-7.

[ref32] Pooch F.; Sliepen M.; Knudsen K. D.; Nyström B.; Tenhu H.; Winnik F. M. Poly(2-isopropyl-2-oxazoline)-b-poly(lactide) (PiPOx-b-PLA) Nanoparticles in Water: Interblock van der Waals Attraction Opposes Amphiphilic Phase Separation. Macromolecules 2019, 52 (3), 1317–1326. 10.1021/acs.macromol.8b02558. 31496543 PMC6727592

[ref33] Yu C.; Li L.; Hu P.; Yang Y.; Wei W.; Deng X.; Wang L.; Tay F. R.; Ma J. Recent Advances in Stimulus-Responsive Nanocarriers for Gene Therapy. Adv. Sci. 2021, 8 (14), 2100540 10.1002/advs.202100540. PMC8292848 34306980

[ref34] Epp-Ducharme B.; Dunne M.; Fan L.; Evans J. C.; Ahmed L.; Bannigan P.; Allen C. Heat-activated nanomedicine formulation improves the anticancer potential of the HSP90 inhibitor luminespib in vitro. Sci. Rep. 2021, 11 (1), 11103 10.1038/s41598-021-90585-w. 34045581 PMC8160139

[ref35] Ridolfo R.; Tavakoli S.; Junnuthula V.; Williams D. S.; Urtti A.; van Hest J. C. M. Exploring the Impact of Morphology on the Properties of Biodegradable Nanoparticles and Their Diffusion in Complex Biological Medium. Biomacromolecules 2021, 22 (1), 126–133. 10.1021/acs.biomac.0c00726. 32510218 PMC7805011

[ref36] Ben-Akiva E.; Meyer R. A.; Yu H.; Smith J. T.; Pardoll D. M.; Green J. J. Biomimetic anisotropic polymeric nanoparticles coated with red blood cell membranes for enhanced circulation and toxin removal. Sci. Adv. 2020, 6 (16), eaay9035 10.1126/sciadv.aay9035. 32490199 PMC7239698

[ref37] Jerca F. A.; Jerca V. V.; Hoogenboom R. In Vitro Assessment of the Hydrolytic Stability of Poly(2-isopropenyl-2-oxazoline). Biomacromolecules 2021, 22 (12), 5020–5032. 10.1021/acs.biomac.1c00994. 34753285

[ref38] Wiesbrock F.; Hoogenboom R.; Leenen M. A. M.; Meier M. A. R.; Schubert U. S. Investigation of the Living Cationic Ring-Opening Polymerization of 2-Methyl-2-Ethyl-2-Nonyl-and 2-Phenyl-2-oxazoline in a Single-Mode Microwave Reactor. Macromolecules 2005, 38 (12), 5025–5034. 10.1021/ma0474170.

[ref39] Weber C.; Becer R. C.; Baumgaertel A.; Hoogenboom R.; Schubert U. S. Preparation of Methacrylate End-Functionalized Poly(2-ethyl-2-oxazoline) Macromonomers. Des. Monomers Polym. 2009, 12 (2), 149–165. 10.1163/156855509X412090.

[ref40] Baumgaertel A.; Weber C.; Knop K.; Crecelius A.; Schubert U. S. Characterization of different poly(2-ethyl-2-oxazoline)s via matrix-assisted laser desorption/ionization time-of-flight tandem mass spectrometry. Rapid Commun. Mass Spectrom. 2009, 23 (6), 756–762. 10.1002/rcm.3933. 19224528

[ref41] Le Fer G.; Le Cœur C.; Guigner J.-M.; Amiel C.; Volet G. Amphiphilic diblock and triblock copolymers based on poly(2-methyl-2-oxazoline) and poly(D,L-lactide): Synthesis, physicochemical characterizations and self-assembly properties. Polymer 2019, 171, 149–160. 10.1016/j.polymer.2019.03.037.

[ref42] Le Fer G.; Le Cœur C.; Guigner J.-M.; Amiel C.; Volet G. Self-assembly of poly(2-alkyl-2-oxazoline-)-g-poly(d,l-lactide) copolymers. Eur. Polym. J. 2017, 88, 656–665. 10.1016/j.eurpolymj.2016.10.008.

[ref43] Wang X.; Li X.; Li Y.; Zhou Y.; Fan C.; Li W.; Ma S.; Fan Y.; Huang Y.; Li N.; et al. Synthesis, characterization and biocompatibility of poly(2-ethyl-2-oxazoline)-poly(d,l-lactide)-poly(2-ethyl-2-oxazoline) hydrogels. Acta Biomater. 2011, 7 (12), 4149–4159. 10.1016/j.actbio.2011.07.011. 21810488

[ref44] Wang C.-H.; Hsiue G.-H. New Amphiphilic Poly(2-ethyl-2-oxazoline)/Poly(l-lactide) Triblock Copolymers. Biomacromolecules 2003, 4 (6), 1487–1490. 10.1021/bm034190s. 14606870

[ref45] Pooch F.; Sliepen M.; Svedström K. J.; Korpi A.; Winnik F. M.; Tenhu H. Inversion of crystallization rates in miscible block copolymers of poly(lactide)-block-poly(2-isopropyl-2-oxazoline). Polym. Chem. 2018, 9 (14), 1848–1856. 10.1039/C8PY00198G.

[ref46] Su F.; Yun P.; Li C.; Li R.; Xi L.; Wang Y.; Chen Y.; Li S. Novel self-assembled micelles of amphiphilic poly(2-ethyl-2-oxazoline) -poly(L-lactide) diblock copolymers for sustained drug delivery. Colloids Surf., A 2019, 566, 120–127. 10.1016/j.colsurfa.2019.01.015.

[ref47] Rettler E. F.-J.; Kranenburg J. M.; Lambermont-Thijs H. M. L.; Hoogenboom R.; Schubert U. S. Thermal, Mechanical, and Surface Properties of Poly(2-N-alkyl-2-oxazoline)s. Macromol. Chem. Phys. 2010, 211 (22), 2443–2448. 10.1002/macp.201000338.

[ref48] Everaerts M.; Tigrine A.; de la Rosa V. R.; Hoogenboom R.; Adriaensens P.; Clasen C.; Van den Mooter G. Unravelling the Miscibility of Poly(2-oxazoline)s: A Novel Polymer Class for the Formulation of Amorphous Solid Dispersions. Molecules 2020, 25 (16), 3587 10.3390/molecules25163587. 32781768 PMC7465563

[ref49] Fox T. G.; Flory P. J. Second-Order Transition Temperatures and Related Properties of Polystyrene. I. Influence of Molecular Weight. J. Appl. Phys. 1950, 21 (6), 581–591. 10.1063/1.1699711.

[ref50] Obeid R.; Tanaka F.; Winnik F. M. Heat-Induced Phase Transition and Crystallization of Hydrophobically End-Capped Poly(2-isopropyl-2-oxazoline)s in Water. Macromolecules 2009, 42 (15), 5818–5828. 10.1021/ma900838v.

[ref51] Oleszko N.; Utrata-Wesołek A.; Wałach W.; Libera M.; Hercog A.; Szeluga U.; Domański M.; Trzebicka B.; Dworak A. Crystallization of Poly(2-isopropyl-2-oxazoline) in Organic Solutions. Macromolecules 2015, 48 (6), 1852–1859. 10.1021/ma502586x.

[ref52] Demirel A. L.; Meyer M.; Schlaad H. Formation of Polyamide Nanofibers by Directional Crystallization in Aqueous Solution. Angew. Chem., Int. Ed. 2007, 46 (45), 8622–8624. 10.1002/anie.200703486. 17907260

[ref53] Jamshidi K.; Hyon S. H.; Ikada Y. Thermal characterization of polylactides. Polymer 1988, 29 (12), 2229–2234. 10.1016/0032-3861(88)90116-4.

[ref54] Kasyapi N.; Bhowmick A. K. Nanolamellar triblock of poly-d,l-lactide-δ-valerolactone-d,l-lactide with tuneable glass transition temperature and crystallinity for use as a drug-delivery vesicle. RSC Adv. 2014, 4 (52), 27439–27451. 10.1039/C4RA02745K.

[ref55] Hoogenboom R.; Thijs H. M. L.; Jochems M. J. H. C.; van Lankvelt B. M.; Fijten M. W. M.; Schubert U. S. Tuning the LCST of poly(2-oxazoline)s by varying composition and molecular weight: alternatives to poly(N-isopropylacrylamide)?. Chem. Commun. 2008, (44), 5758–5760. 10.1039/b813140f. 19009072

[ref56] Park J.-S.; Kataoka K. Precise Control of Lower Critical Solution Temperature of Thermosensitive Poly(2-isopropyl-2-oxazoline) via Gradient Copolymerization with 2-Ethyl-2-oxazoline as a Hydrophilic Comonomer. Macromolecules 2006, 39 (19), 6622–6630. 10.1021/ma0605548.

[ref57] Seifert U.; Berndl K.; Lipowsky R. Shape transformations of vesicles: Phase diagram for spontaneous- curvature and bilayer-coupling models. Phys. Rev. A: At., Mol., Opt. Phys. 1991, 44 (2), 1182–1202. 10.1103/PhysRevA.44.1182. 9906067

[ref58] Ashjari M.; Kazemi M.; Abi M. N.; Mohammadi M.; Rafiezadeh S. Poly (isopropyl-oxazoline) micelle nanocarrier as dual-responsive prodrug for targeted doxorubicin delivery. J. Drug Delivery Sci. Technol. 2020, 59, 101914 10.1016/j.jddst.2020.101914.

[ref59] Chen W.; Su L.; Zhang P.; Li C.; Zhang D.; Wu W.; Jiang X. Thermo and pH dual-responsive drug-linked pseudo-polypeptide micelles with a comb-shaped polymer as a micellar exterior. Polym. Chem. 2017, 8 (44), 6886–6894. 10.1039/C7PY01389B.

[ref60] Akbar M.; Cagli E.; Erel-Göktepe I. Layer-By-Layer Modified Superparamagnetic Iron Oxide Nanoparticles with Stimuli-Responsive Drug Release Properties. Macromol. Chem. Phys. 2019, 220 (4), 1800422 10.1002/macp.201800422.

[ref61] Toncheva-Moncheva N.; Veleva-Kostadinova E.; Tsvetanov C.; Momekova D.; Rangelov S. Preparation and properties of positively charged mesoglobules based on poly(2-isopropyl-2-oxazoline) and evaluation of their potential as carriers of polynucleotides. Polymer 2017, 111, 156–167. 10.1016/j.polymer.2017.01.049.

[ref62] Zhu J.; Gong Z.; Yang C.; Yan Q. Reshaping Membrane Polymorphism of Polymer Vesicles through Dynamic Gas Exchange. J. Am. Chem. Soc. 2021, 143 (48), 20183–20191. 10.1021/jacs.1c07838. 34813319

[ref63] Salva R.; Le Meins J.-F.; Sandre O.; Brûlet A.; Schmutz M.; Guenoun P.; Lecommandoux S. Polymersome Shape Transformation at the Nanoscale. ACS Nano 2013, 7 (10), 9298–9311. 10.1021/nn4039589. 24047230

[ref64] Men Y.; Li W.; Tu Y.; Peng F.; Janssen G.-J. A.; Nolte R. J. M.; Wilson D. A. Nonequilibrium Reshaping of Polymersomes via Polymer Addition. ACS Nano 2019, 13 (11), 12767–12773. 10.1021/acsnano.9b04740. 31697471 PMC6887890

[ref65] Rikken R. S. M.; Engelkamp H.; Nolte R. J. M.; Maan J. C.; van Hest J. C. M.; Wilson D. A.; Christianen P. C. M. Shaping polymersomes into predictable morphologies via out-of-equilibrium self-assembly. Nat. Commun. 2016, 7 (1), 12606 10.1038/ncomms12606. 27558520 PMC5007325

[ref66] Legros C.; De Pauw-Gillet M.-C.; Tam K. C.; Taton D.; Lecommandoux S. Crystallisation-driven self-assembly of poly(2-isopropyl-2-oxazoline)-block-poly(2-methyl-2-oxazoline) above the LCST. Soft Matter 2015, 11 (17), 3354–3359. 10.1039/C5SM00313J. 25793873

[ref67] Meyer M.; Antonietti M.; Schlaad H. Unexpected thermal characteristics of aqueous solutions of poly(2-isopropyl-2-oxazoline). Soft Matter 2007, 3 (4), 430–431. 10.1039/b616678d. 32900061

[ref68] Men Y.; Li W.; Janssen G.-J.; Rikken R. S. M.; Wilson D. A. Stomatocyte in Stomatocyte: A New Shape of Polymersome Induced via Chemical-Addition Methodology. Nano Lett. 2018, 18 (3), 2081–2085. 10.1021/acs.nanolett.8b00187. 29411614 PMC5997403

[ref69] Góźdź W. T. The Interface Width of Separated Two-Component Lipid Membranes. J. Phys. Chem. B 2006, 110 (43), 21981–21986. 10.1021/jp062304z. 17064167

[ref70] Spiers L.; Gray M.; Lyon P.; Sivakumar S.; Bekkali N.; Scott S.; Collins L.; Carlisle R.; Wu F.; Middleton M.; et al. Clinical trial protocol for PanDox: a phase I study of targeted chemotherapy delivery to non-resectable primary pancreatic tumours using thermosensitive liposomal doxorubicin (ThermoDox) and focused ultrasound. BMC Cancer 2023, 23 (1), 896 10.1186/s12885-023-11228-z. 37741968 PMC10517508

[ref71] Dunne M.; Regenold M.; Allen C. Hyperthermia can alter tumor physiology and improve chemo- and radio-therapy efficacy. Adv. Drug Delivery Rev. 2020, 163–164, 98–124. 10.1016/j.addr.2020.07.007. 32681862

