# Supplementary material for: Poly(2-oxazoline)-Based Thermoresponsive Stomatocytes
Source: Biomacromolecules. 2024 Aug 15;25(9):6050–9. doi: 10.1021/acs.biomac.4c00726 (PMC11388456; doi:10.1021/acs.biomac.4c00726)
Supplement: Supplementary file 1 — bm4c00726_si_001.pdf [file bm4c00726_si_001.pdf]

Electronic Supporting Information

Poly(2-oxazoline) based Thermoresponsive Stomatocytes

Roberto Terracciano [a], Yuechi Liu [b], Zivani Varanaraja [a], Magdalena Godzina [a], Gokhan Yilmaz [a], Jan C. M. van Hest [b*] and C. Remzi Becer [a*]

[a] *Department of Chemistry, University of Warwick, Coventry, CV4 7AL, United Kingdom*

[b] *Eindhoven University of Technology, P.O. Box 513 (STO 3.31), 5600MB Eindhoven, The Netherlands*

* *Corresponding author:* j.c.m.v.hest@tue.nl, Remzi.Becer@warwick.ac.uk

Synthesis of POx Homopolymers

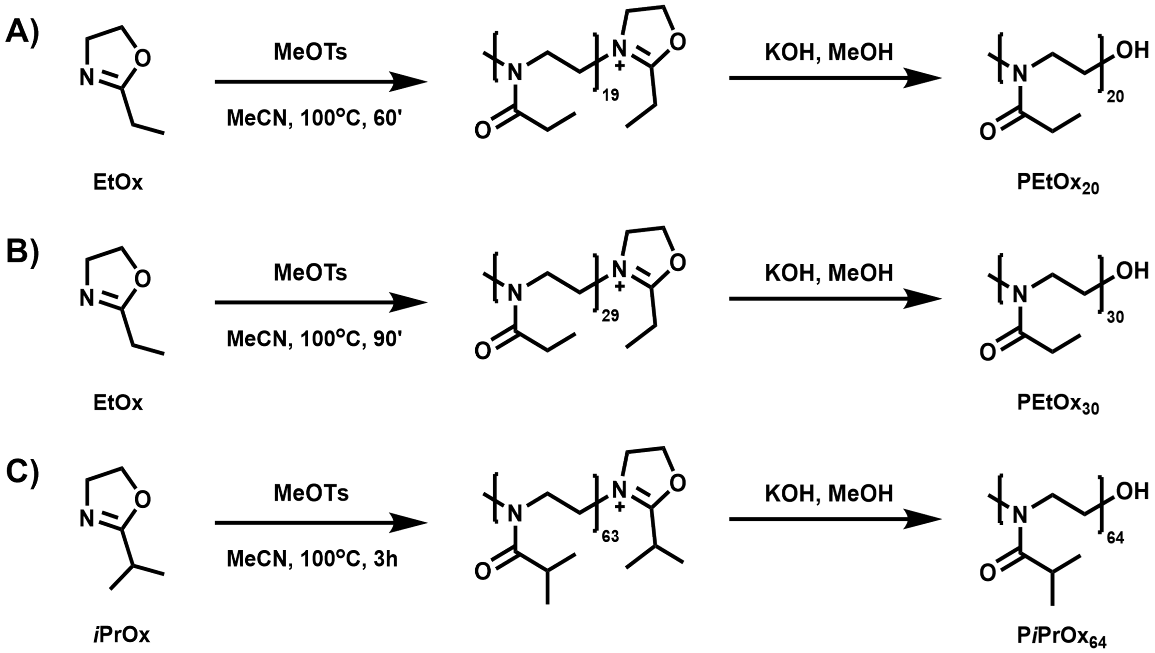

Scheme S1. (A) Schematic synthesis of hydroxyl-terminated $PEtOx_{20}$, (B) $PEtOx_{30}$ and (C) P*i*$PrOx_{64}$ by cationic ring opening polymerization (CROP) using 2-ethyl-2-oxazoline and 2-isopropyl-2-oxazoline monomers, and methyl p-toluenesulfonate as initiator. All the polymers are obtained by direct endcapping after CROP with potassium hydroxide.

Synthesis of POx_n-$PDLLA_m$ diblock copolymers

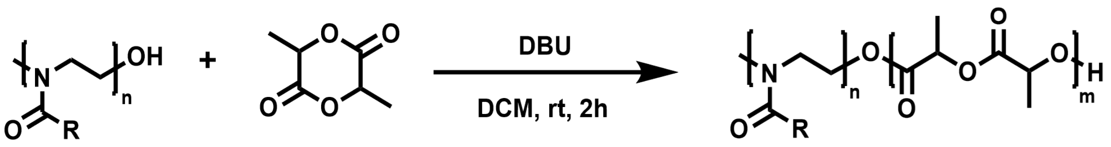

Scheme S2. (B) General schematic synthesis of POx_n-*b*-$PDLLA_m$ diblock copolymer by ring opening polymerization (ROP) using hydroxyl-terminated POx as macroinitiator, d,l-lactide monomer, and DBU as catalyst.

Supplementary Figures

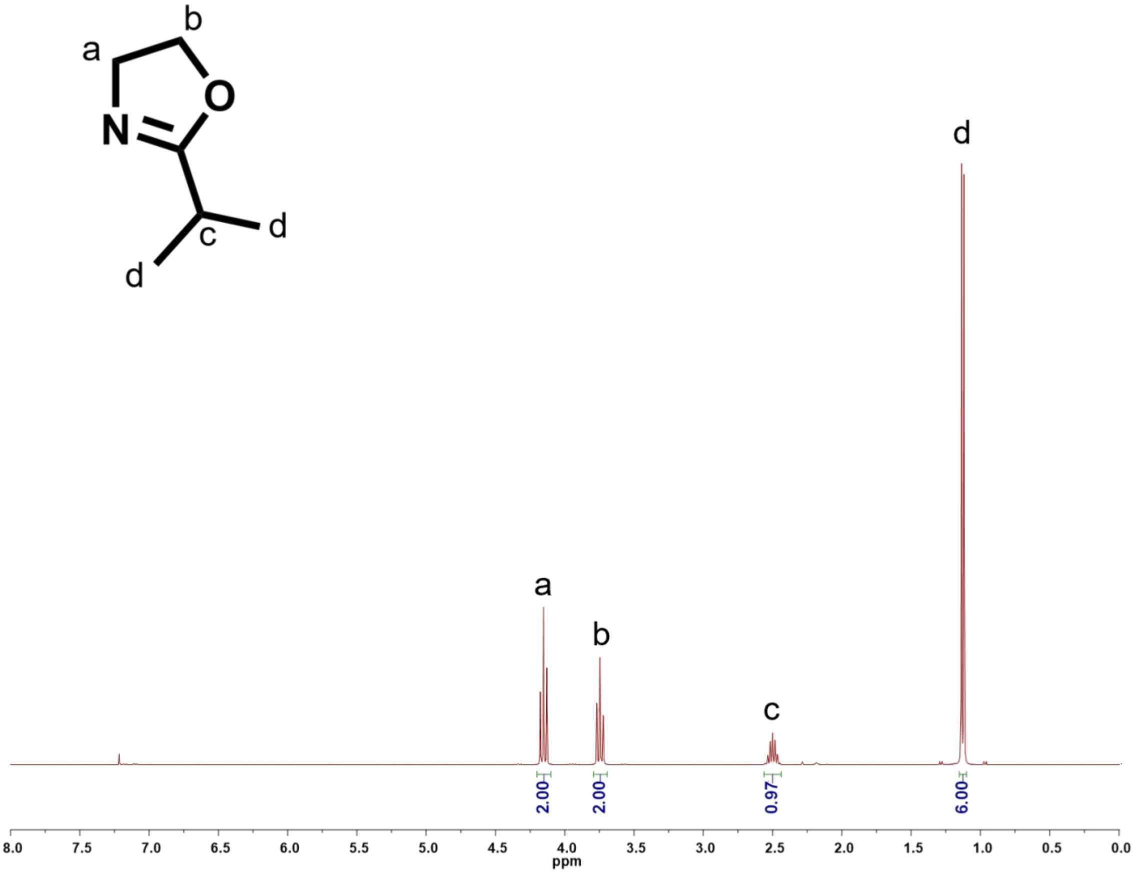

Figure S1. ^1H NMR of 2-isopropyl-2-oxazoline in $CDCl_3$.

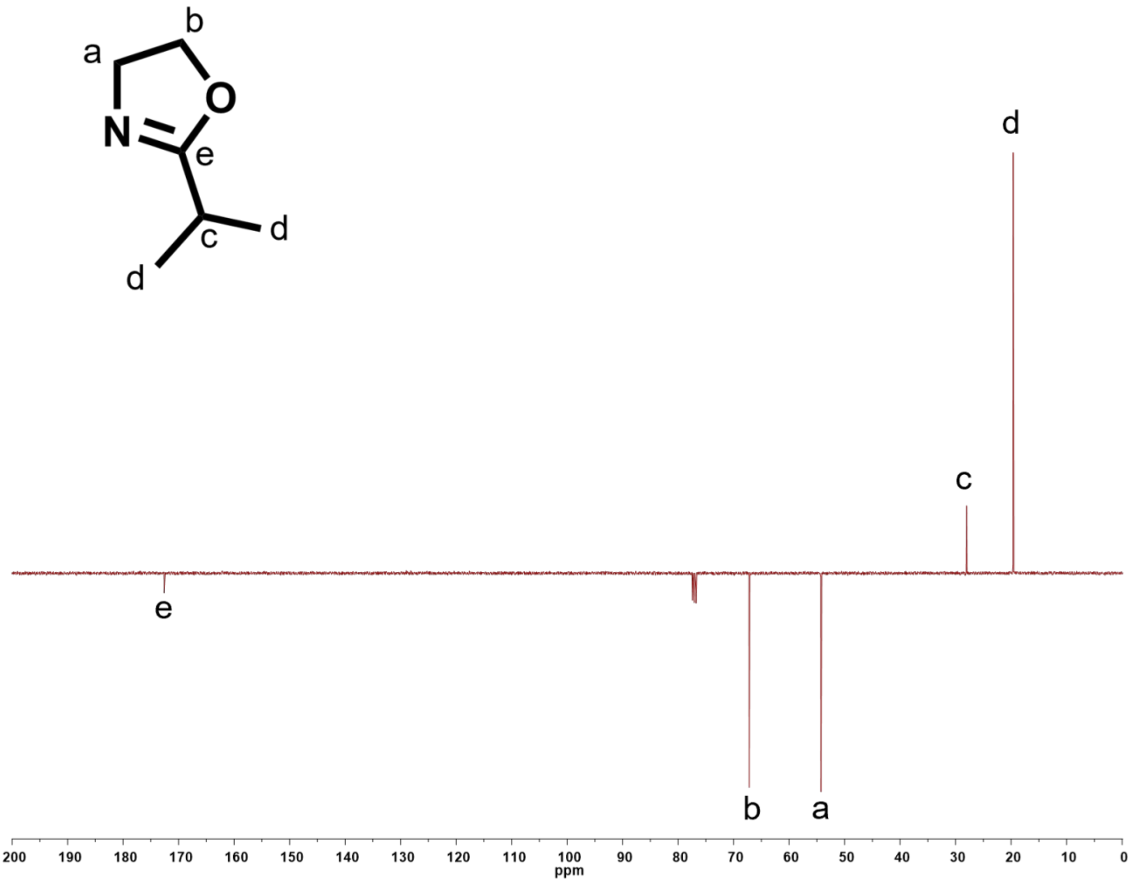

Figure S2. ^{13}C NMR of 2-isopropyl-2-oxazoline in $CDCl_{3.}$

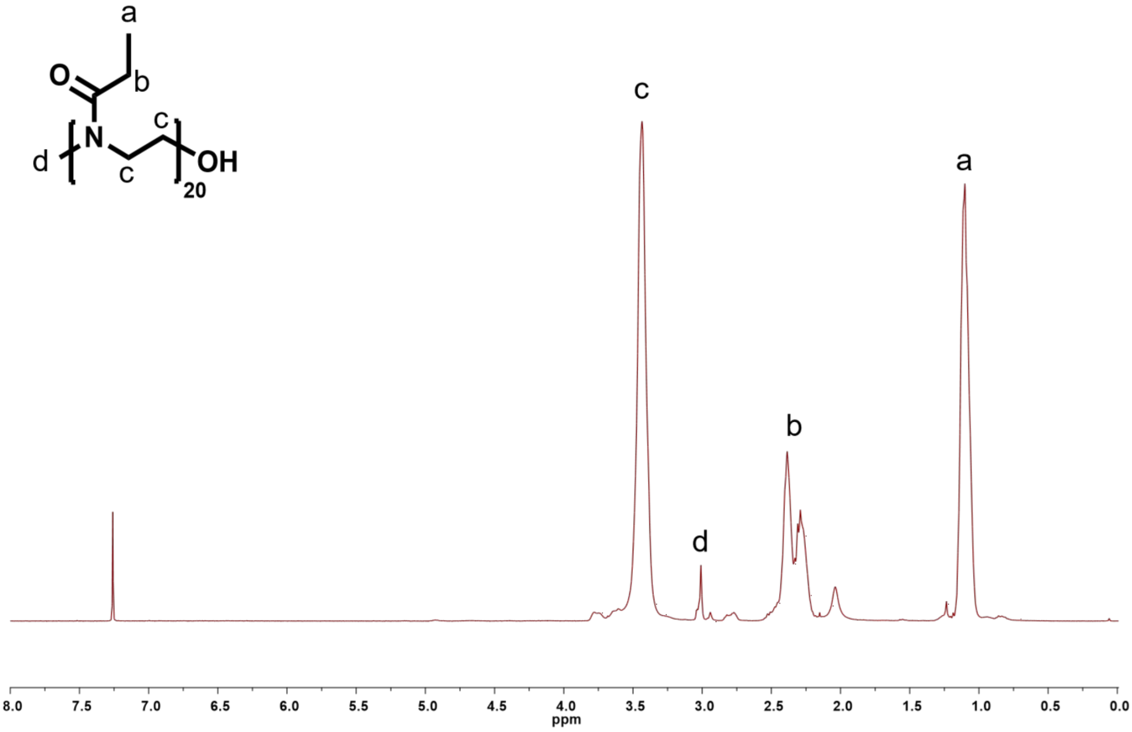

Figure S3. ^{1}H NMR of $PEtOx_{20}$ after end-capping and purification in $CDCl_{3.}$

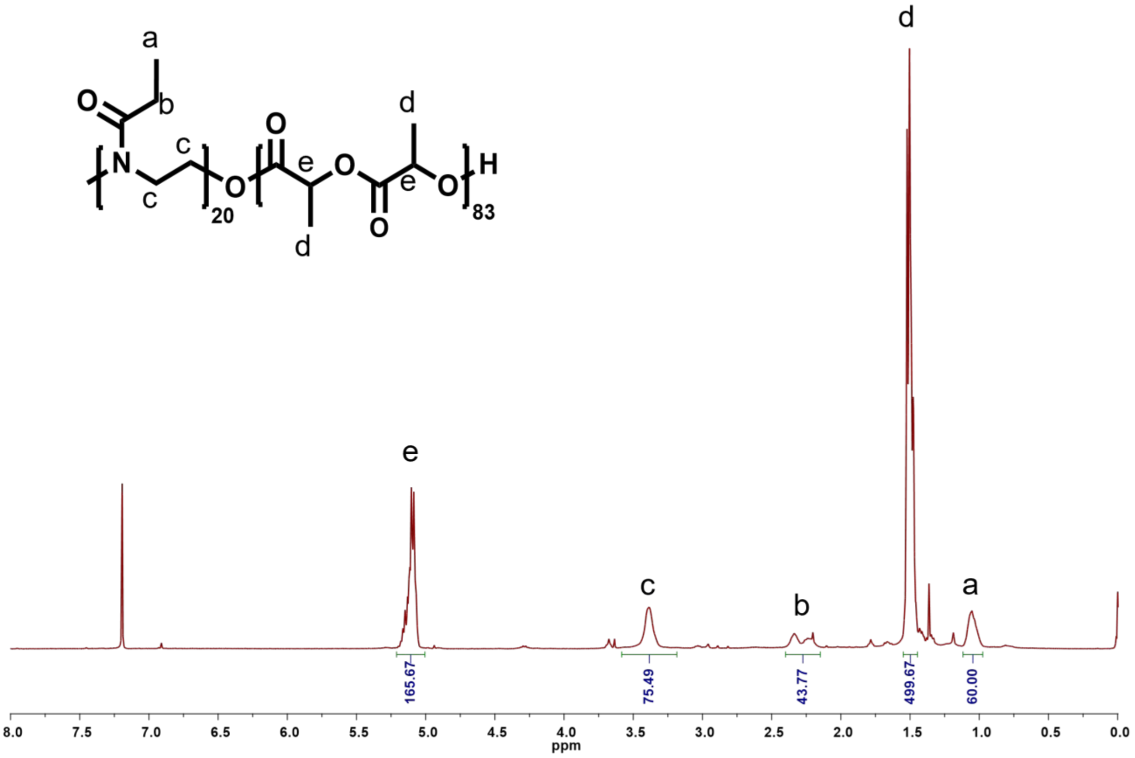

Figure S4. ^{1}H NMR of $PEtOx_{20}$-*b*-$PDLLA_{83}$ in $CDCl_3$.

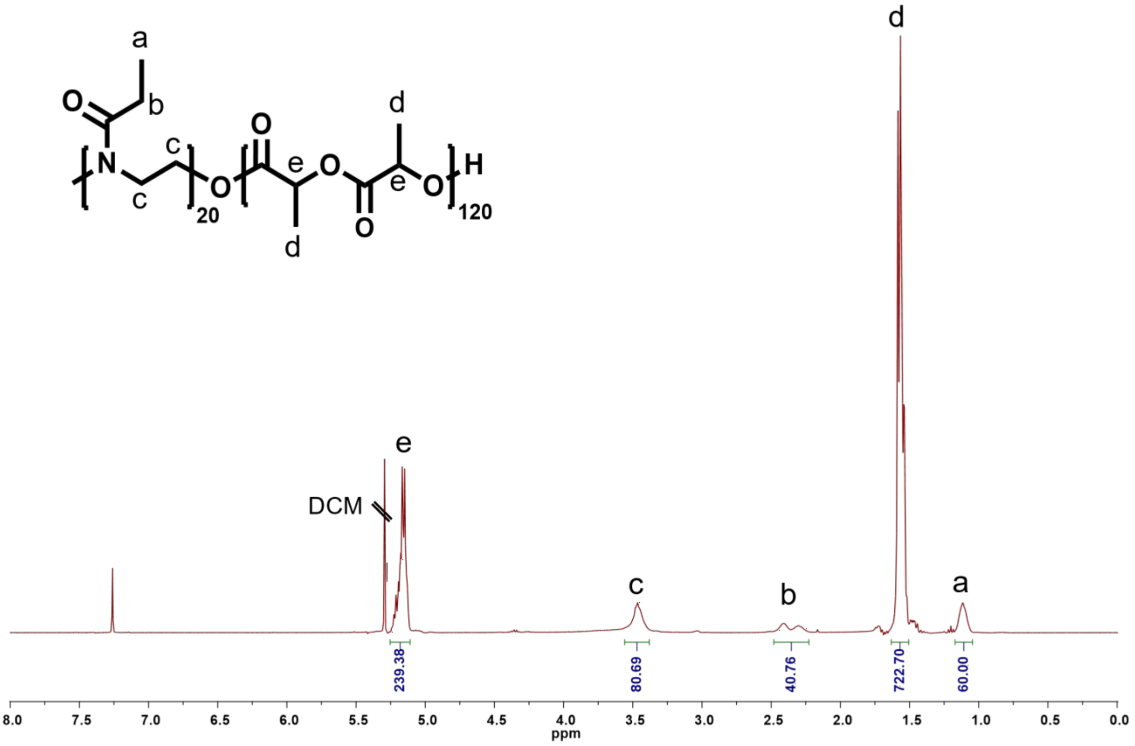

Figure S5. ^{1}H NMR of $PEtOx_{20}$-*b*-$PDLLA_{120}$ in $CDCl_3$.

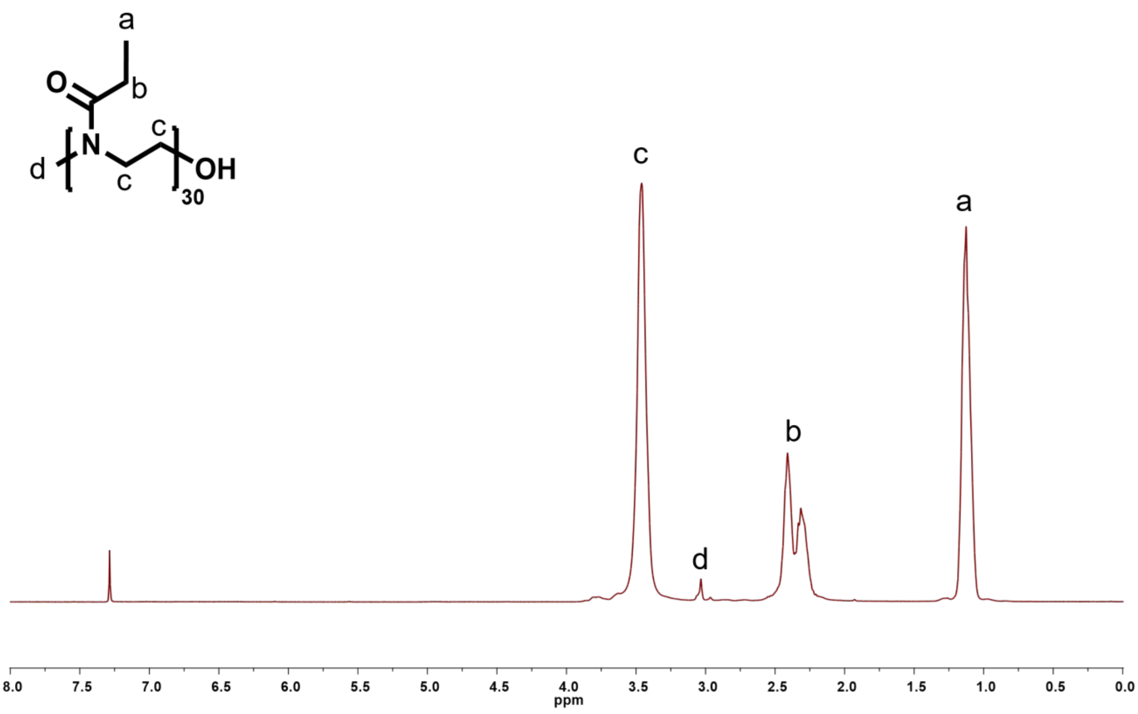

Figure S6. ^{1}H NMR of $PEtOx_{30}$ after end-capping and purification in $CDCl_3$.

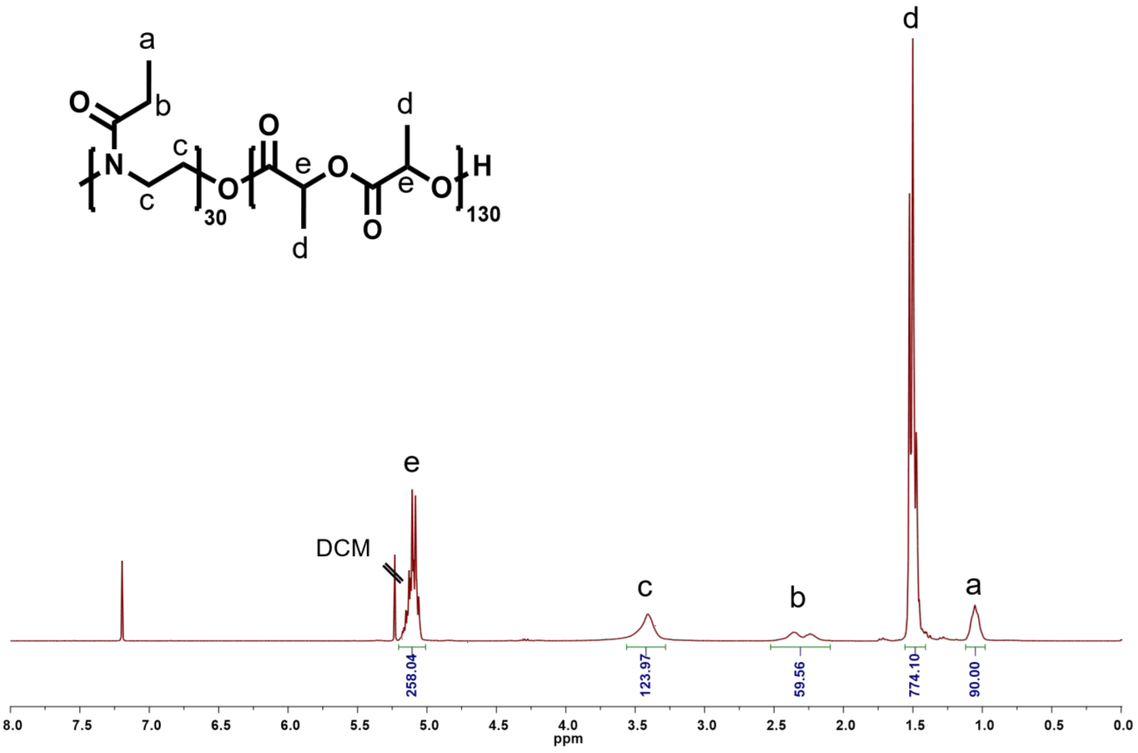

Figure S7. ^{1}H NMR of $PEtOx_{30}$-*b*-$PDLLA_{130}$ in $CDCl_3$.

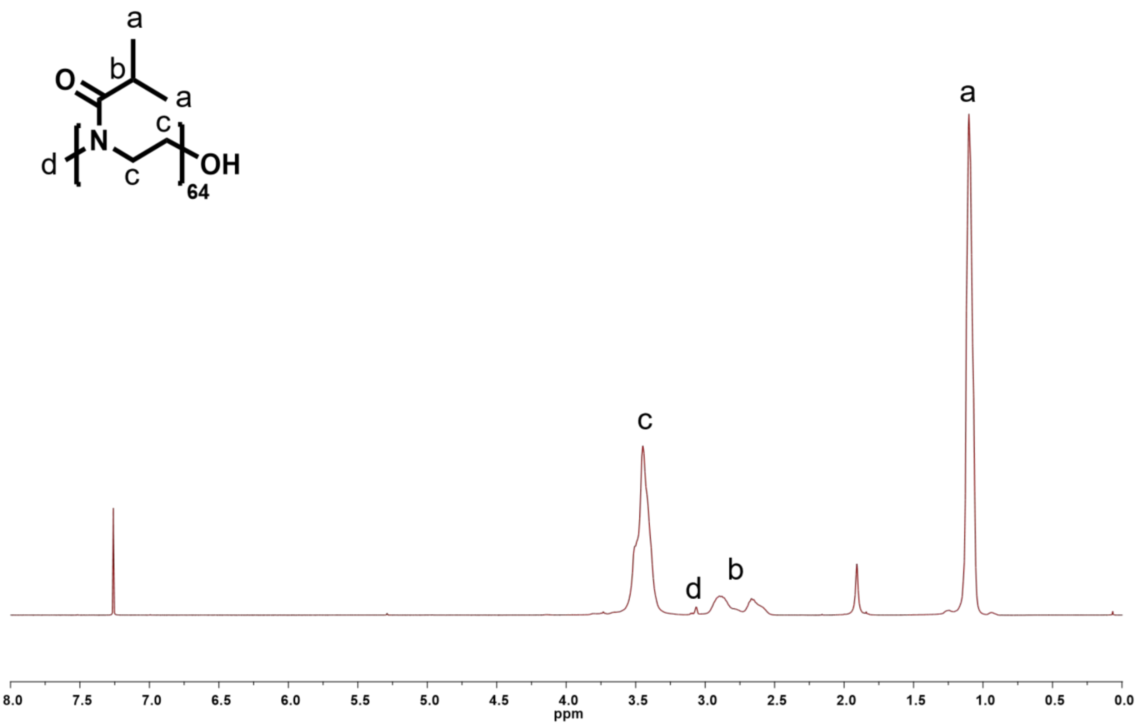

Figure S8. ^{1}H NMR of P*i*PrOx$_{64}$ after end-capping and purification in $CDCl_3$.

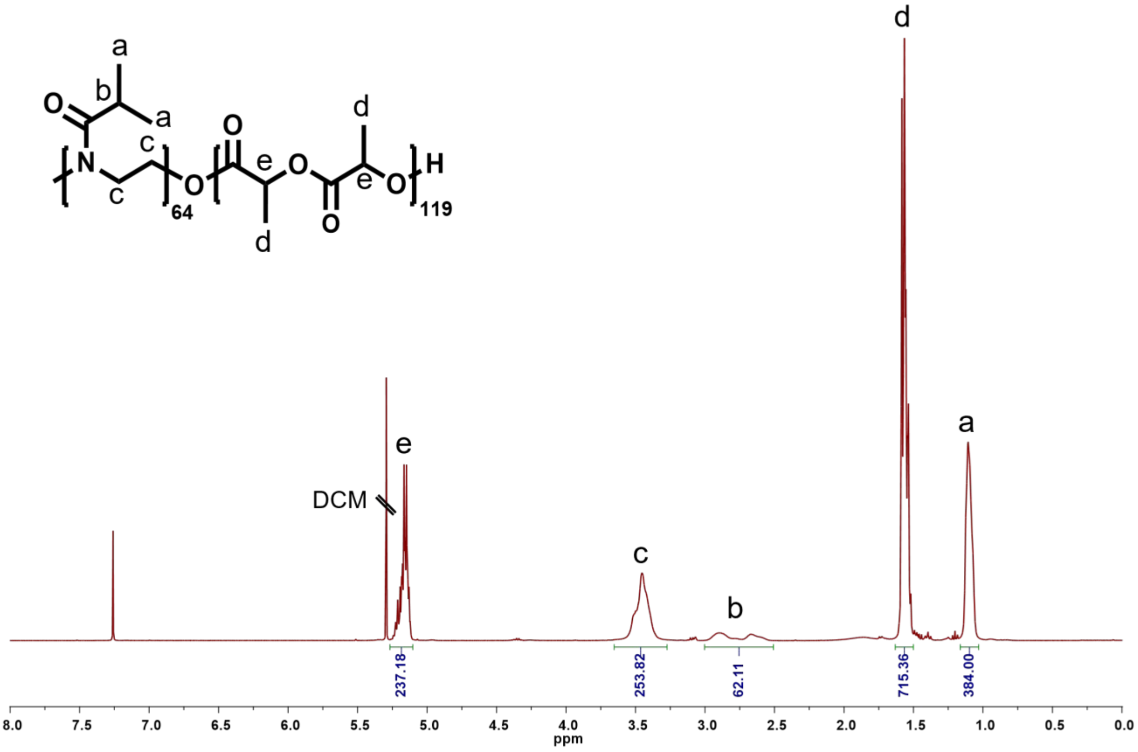

Figure S9. ^{1}H NMR of P*i*PrOx$_{64}$-*b*-PDLLA$_{119}$ in $CDCl_3$.

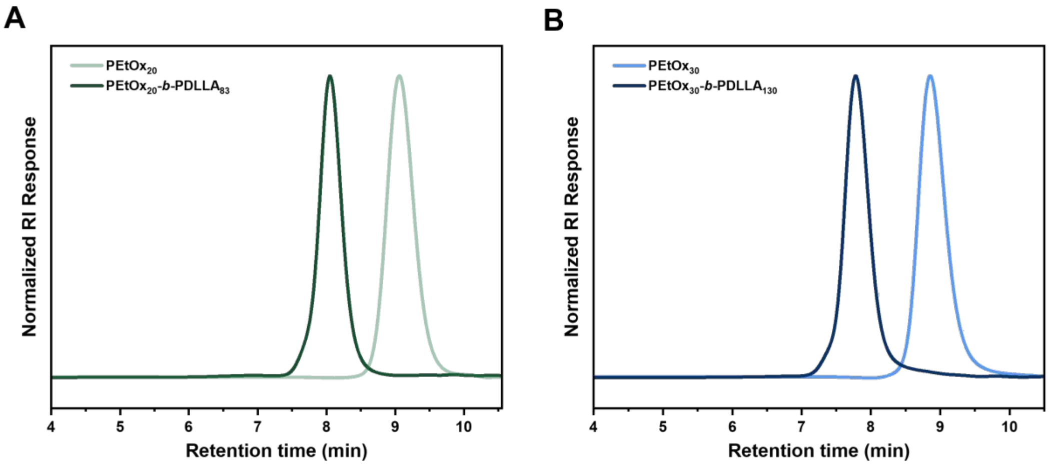

Figure S10. GPC-SEC traces of (A) $PEtOx_{20}$ and (B) $PEtOx_{30}$, along with their respective $PEtOx_{20}$-$PDLLA_{83}$ and $PEtOx_{30}$-$PDLLA_{130}$ diblock copolymers.

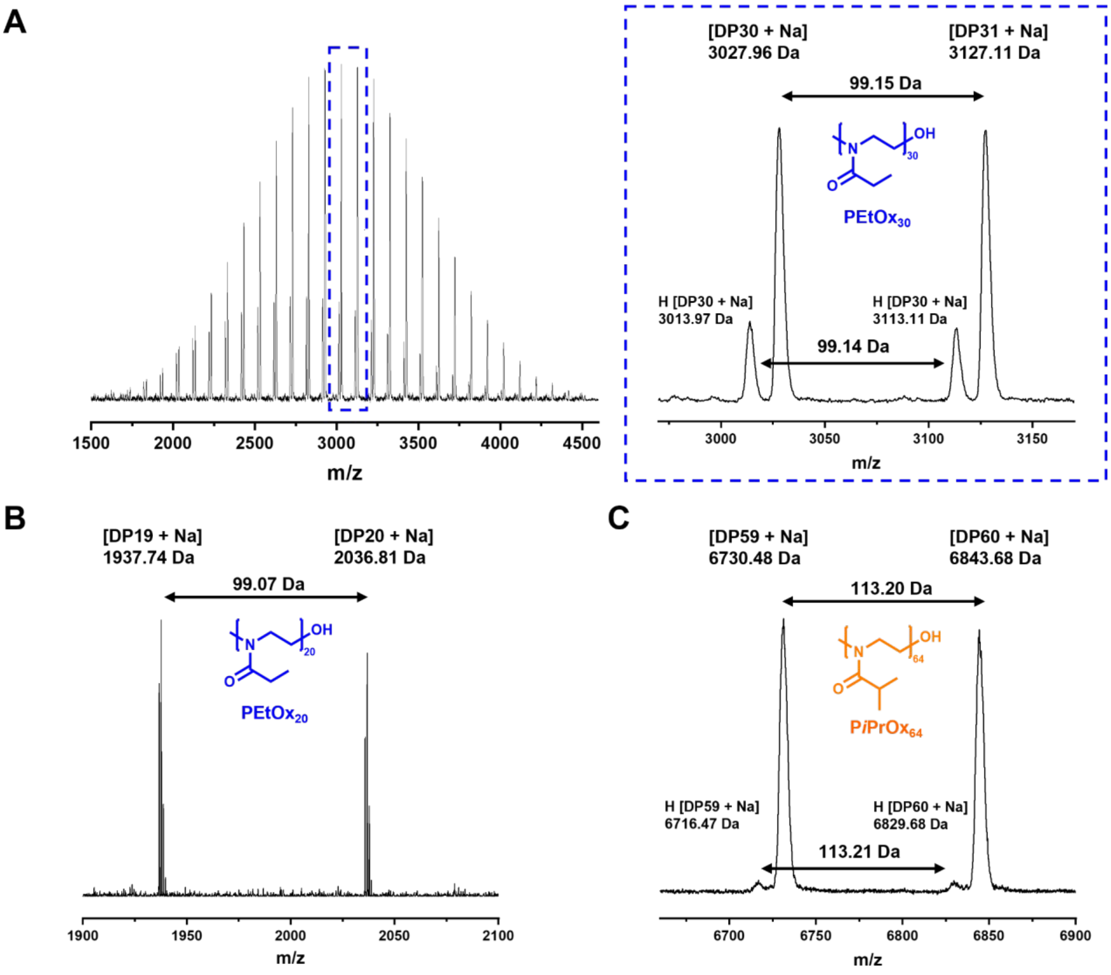

Figure S11. (A) Full MALDI-ToF spectrum and enlarged inset (dashed box) of $PEtOx_{30}$. (B) Enlarged MALDI-ToF spectra of $PEtOx_{20}$ and (C) P*i*$PrOx_{64}$. The distributions correspond to the sodium adduct of the hydroxy-terminated polymers.

Table S1. Found and calculated m/z values of poly(2-oxazoline) homopolymers from the MALDI-ToF Spectra in Fig. 2 and Fig. S11.

Sample	[M + Na] found (Da)	[M + Na] calculated (Da)	Difference (Da)
$PEtOx_{20}$	2036.81	2036.68	0.13
$PEtOx_{30}$	3027.96	3028.01	0.05
P*i*$PrOx_{64}$	6843.68	6843.62	0.06

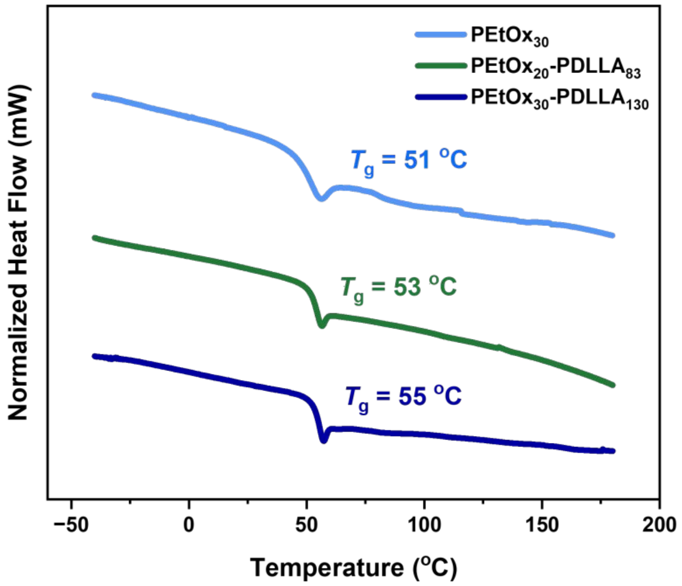

Figure S12. Differential scanning calorimetry (DSC) thermograms of the second heating cycle of hydroxy-terminated $PEtOx_{30}$ homopolymer, and $PEtOx_{20}$-$PDLLA_{83}$ and $PEtOx_{30}$-$PDLLA_{130}$ diblock copolymers. Glass transition temperatures were calculated from the inflection point of the curves.

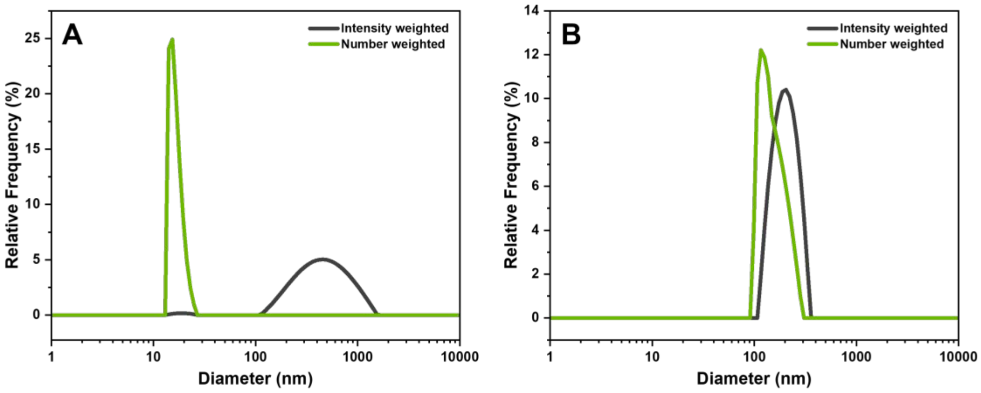

Figure S13. Intensity and number weighted DLS traces of PEtOx$_{20}$-*b*-PDLLA$_{83}$ nanoparticle size (A) before and (B) after dialysis.

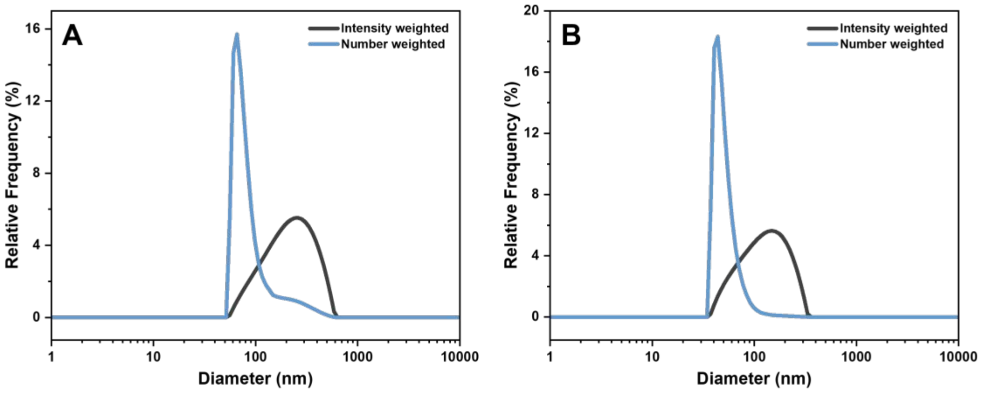

Figure S14. Intensity and number weighted DLS traces of PEtOx$_{30}$-*b*-PDLLA$_{130}$ nanoparticle size (A) before and (B) after dialysis.

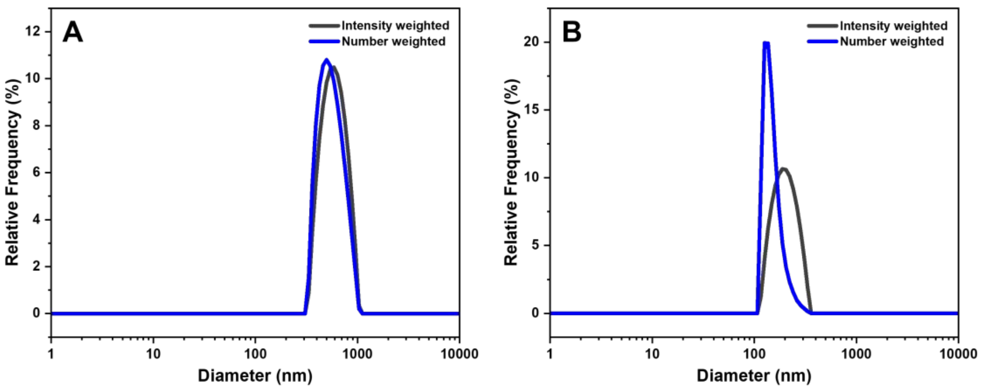

Figure S15. Intensity and number weighted DLS traces of PEtOx$_{20}$-*b*-PDLLA$_{120}$ nanoparticle size (A) before and (B) after dialysis.

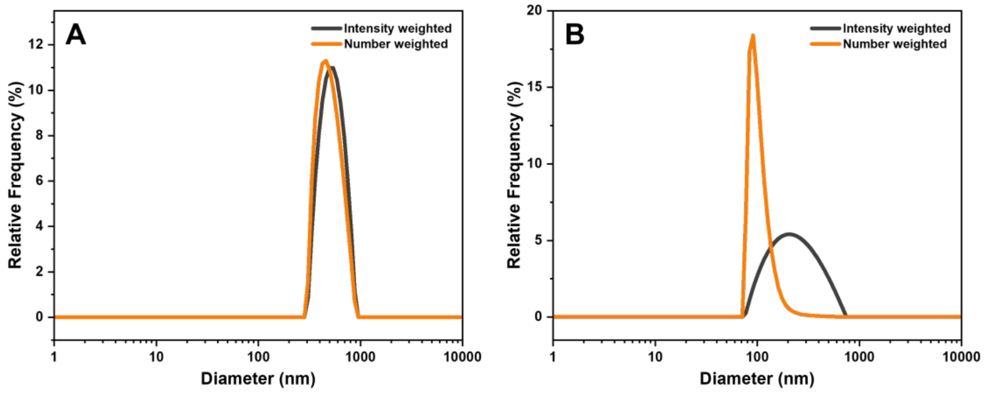

Figure S16. Intensity and number weighted DLS traces of PEtOx$_{20}$-*b*-PDLLA$_{120}$ and P*i*PrOx$_{64}$-*b*-PDLLA$_{119}$ nanoparticle size (A) before and (B) after dialysis.

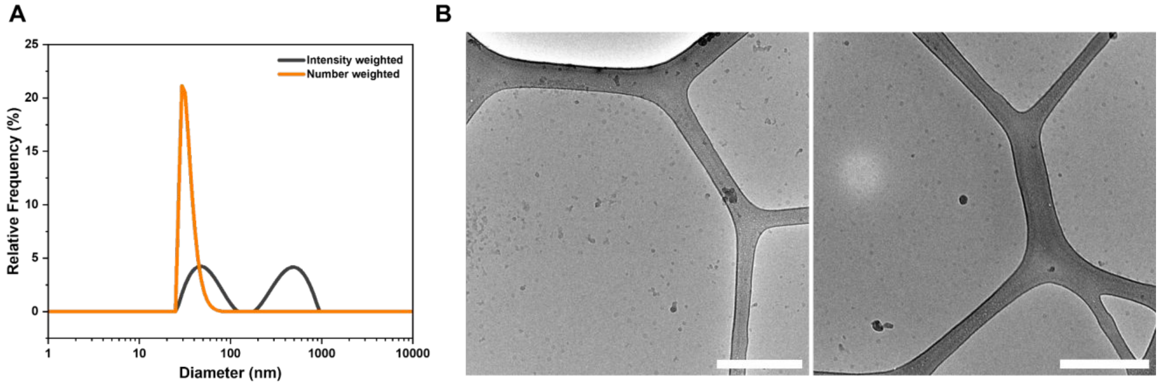

Figure S17. (A) Intensity and number weighted DLS traces of P*i*PrOx$_{64}$-*b*-PDLLA$_{119}$ nanoparticle size. (B) Cryo-TEM images showing micelles of ~ 50 nm and aggregates formation from P*i*PrOx$_{64}$-*b*-PDLLA$_{119}$. Scale bars correspond to 400 nm.

Table S2. Number averaged sizes of POx-PDLLA formulations before and after dialysis against a saline solution (75 mM NaCl) at 5 mg/mL obtained by DLS.

Sample	Average Diameter (nm) Before dialysis	PDI	Average Diameter (nm) After dialysis	PDI
PEtOx$_{20}$-*b*-PDLLA$_{83}$	438	0.22	196	0.13
PEtOx$_{30}$-*b*-PDLLA$_{130}$	203	0.26	115	0.25
PEtOx$_{20}$-*b*-PDLLA$_{120}$	594	0.09	202	0.09
PEtOx$_{20}$-*b*-PDLLA$_{120}$ /P*i*PrOx$_{64}$-*b*-PDLLA$_{119}$	587	0.18	220	0.19
P*i*PrOx$_{64}$-*b*-PDLLA$_{119}$	231	0.30	-	-

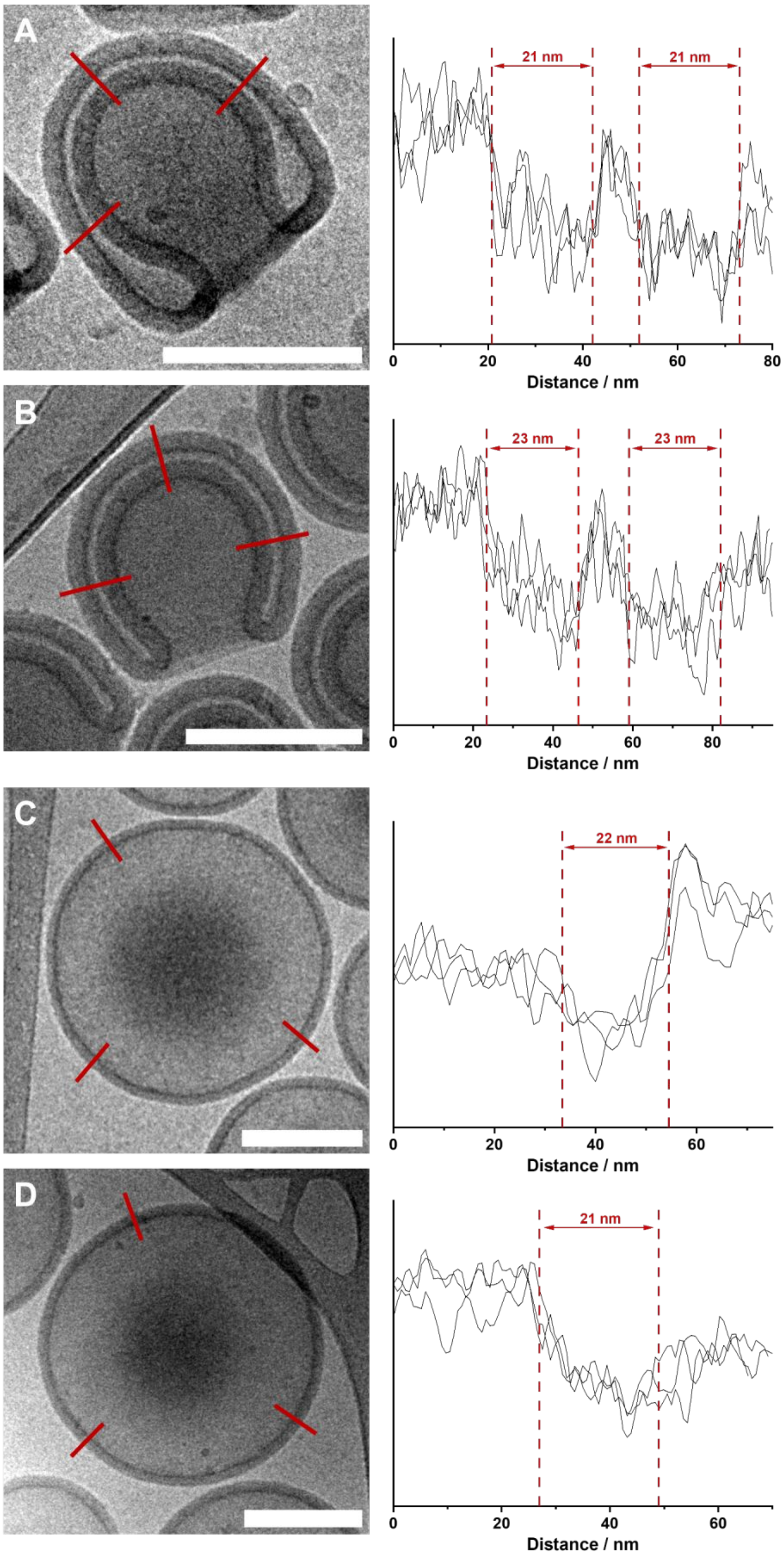

Figure S18. Cryo-TEM images of stomatocytes and polymersomes with varying membrane thickness and corresponding line profile: (A) PEtOx$_{20}$-*b*-PDLLA$_{120}$ stomatocytes, (B) PEtOx$_{20}$-*b*-PDLLA$_{120}$ and P*i*PrOx$_{64}$-*b*-PDLLA$_{119}$ stomatocytes, (C) PEtOx$_{20}$-*b*-PDLLA$_{120}$ polymersomes, and (D) PEtOx$_{20}$-*b*-PDLLA$_{120}$ and P*i*PrOx$_{64}$-*b*-PDLLA$_{119}$ polymersomes. All scale bars correspond to 200 nm.

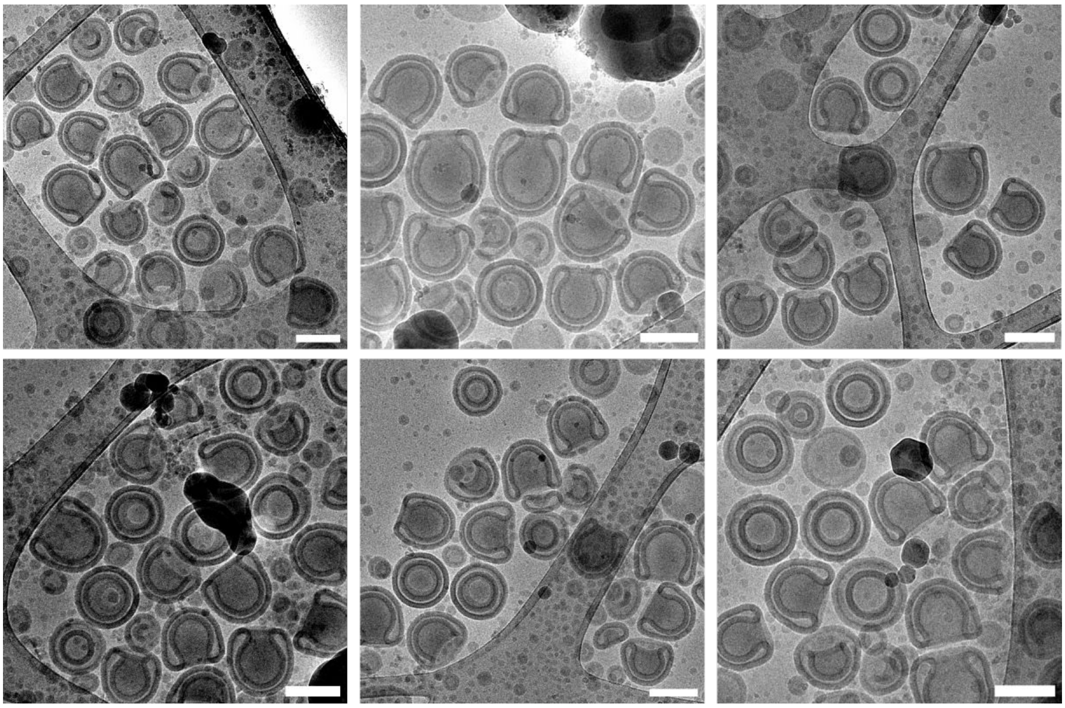

Figure S19. Cryo-TEM images of PEtOx$_{20}$-*b*-PDLLA$_{120}$ stomatocytes. All scale bars correspond to 200 nm.

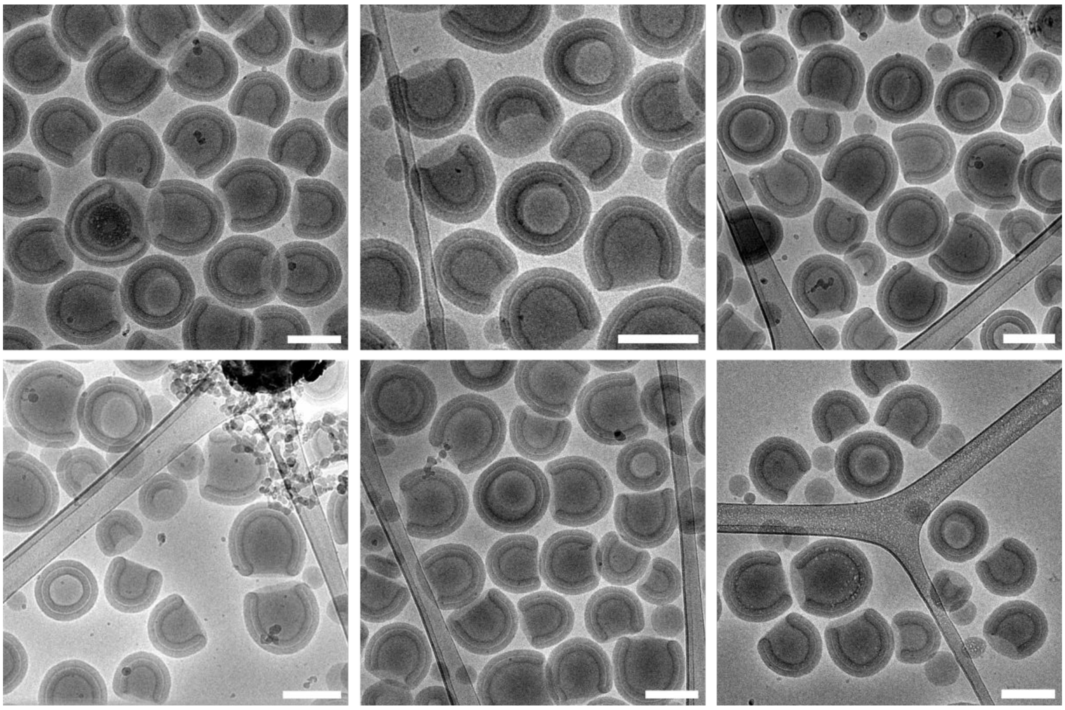

Figure S20. Cryo-TEM images of PEtOx$_{20}$-*b*-PDLLA$_{120}$ and PEtOx$_{20}$-*b*-PDLLA$_{120}$ and P*i*PrOx$_{64}$-*b*-PDLLA$_{119}$ stomatocytes. All scale bars correspond to 200 nm.

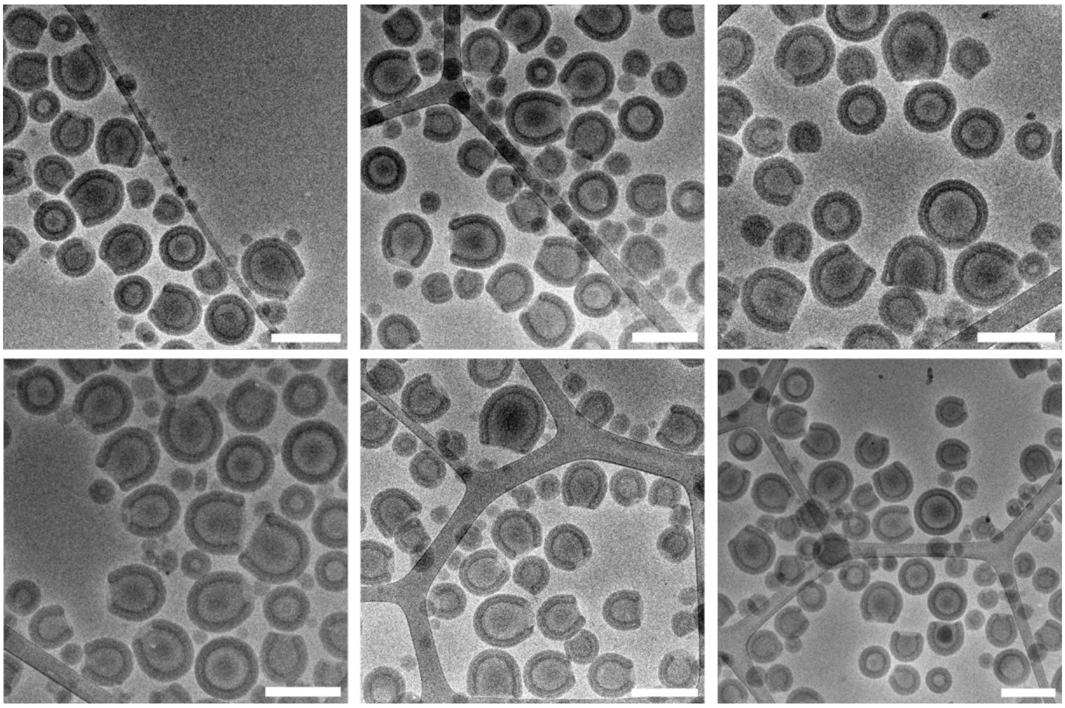

Figure S21. Cryo-TEM images of PEtOx$_{20}$-*b*-PDLLA$_{120}$ stomatocytes after thermal exposure at 42 ℃ at a concentration of 10 mg/ml in Milli-Q water (150 mM NaCl). All scale bars correspond to 400 nm.

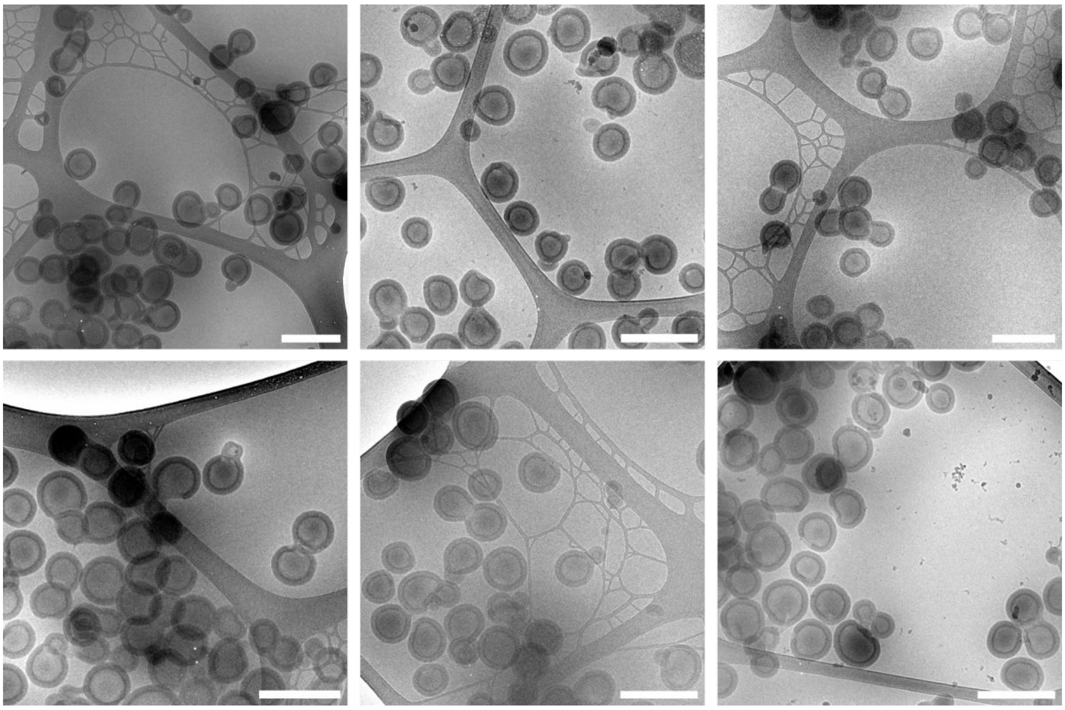

Figure S22. Cryo-TEM images of PEtOx$_{20}$-*b*-PDLLA$_{120}$ and P*i*PrOx$_{64}$-*b*-PDLLA$_{119}$ stomatocytes after thermal exposure at 40-42 ℃ at a concentration of 10 mg/ml in Milli-Q water (150 mM NaCl). All scale bars correspond to 500 nm.

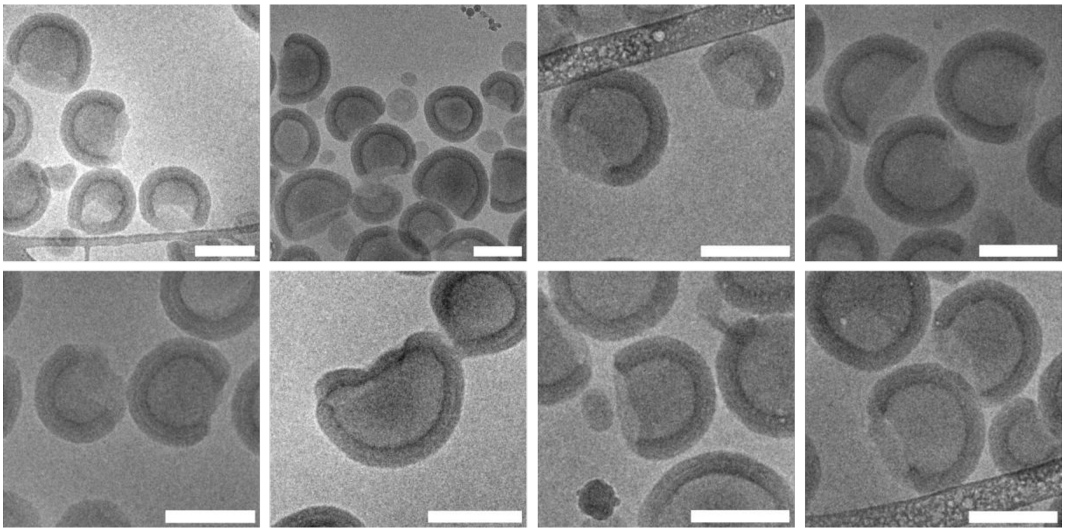

Figure S23. Cryo-TEM images of PEtOx$_{20}$-*b*-PDLLA$_{120}$ and P*i*PrOx$_{64}$-*b*-PDLLA$_{119}$ stomatocytes after thermal exposure at 40-42 °C, depicting single stomatocytes with characteristic "wobbly edges". All scale bars correspond to 200 nm.

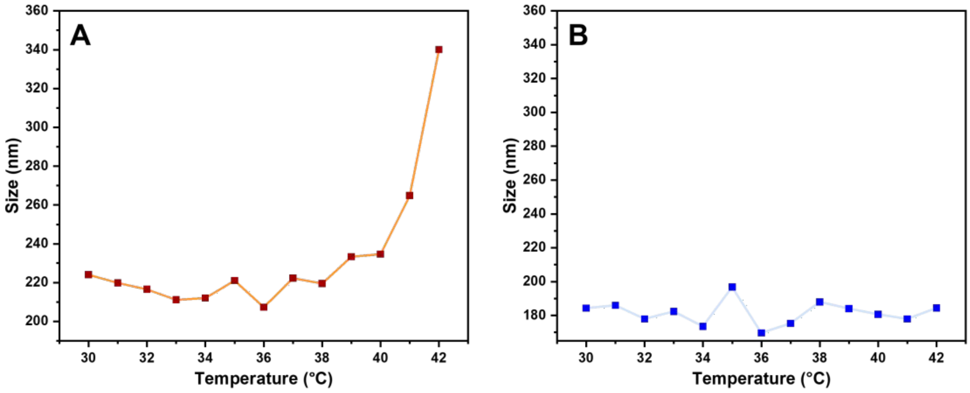

Figure S24. Temperature-dependent DLS of (A) PEtOx$_{20}$-*b*-PDLLA$_{120}$ and P*i*PrOx$_{64}$-*b*-PDLLA$_{119}$ stomatocytes and (B) PEtOx$_{20}$-*b*-PDLLA$_{120}$ stomatocytes at a concentration of 10 mg/ml in Milli-Q water (150 mM NaCl).

References

(1) Jerca, F. A.; Jerca, V. V.; Hoogenboom, R. In Vitro Assessment of the Hydrolytic Stability of Poly(2-isopropenyl-2-oxazoline). *Biomacromolecules* **2021**, *22* (12), 5020-5032.

(2) Abdelmohsen, L. K. E. A.; Williams, D. S.; Pille, J.; Ozel, S. G.; Rikken, R. S. M.; Wilson, D. A.; van Hest, J. C. M. Formation of Well-Defined, Functional Nanotubes via Osmotically Induced Shape Transformation of Biodegradable Polymersomes. *Journal of the American Chemical Society* **2016**, *138* (30), 9353-9356.

(3) Pijpers, I. A. B.; Abdelmohsen, L. K. E. A.; Williams, D. S.; van Hest, J. C. M. Morphology Under Control: Engineering Biodegradable Stomatocytes. *ACS Macro Letters* **2017**, *6* (11), 1217-1222.
